# Origin of Catalysis and Selectivity in Lewis Acid-Promoted Diels–Alder Reactions Involving Vinylazaarenes as Dienophiles

**DOI:** 10.1021/acs.joc.2c01035

**Published:** 2022-07-07

**Authors:** Susana Portela, Israel Fernández

**Affiliations:** Departamento de Química Orgánica I and Centro de Innovación en Química Avanzada (ORFEO-CINQA), Facultad de Ciencias Químicas, Universidad Complutense de Madrid, 28040 Madrid, Spain

## Abstract

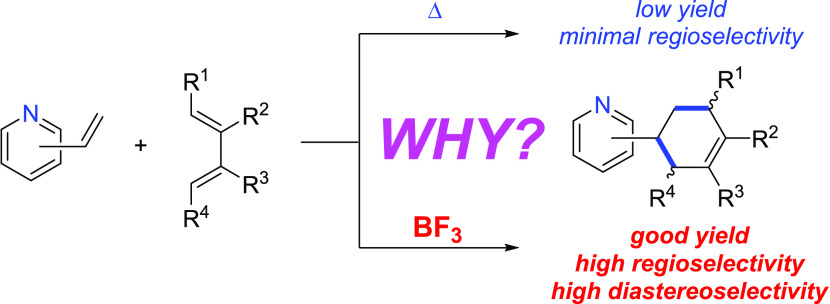

The poorly understood factors controlling the catalysis and selectivity in Lewis acid-promoted Diels–Alder cycloaddition reactions involving vinylazaarenes as dienophiles have been quantitatively explored in detail by means of computational methods. With the help of the activation strain model and the energy decomposition analysis methods, it is found that the remarkable acceleration induced by the catalysis is mainly due to a significant reduction of the Pauli repulsion between the key occupied π-molecular orbitals of the reactants and not due to the proposed stabilization of the lowest unoccupied molecular orbital (LUMO) of the dienophile. This computational approach has also been helpful to understand the reasons behind the extraordinary regio- and diastereoselectivity observed experimentally. The insight gained in this work allows us to predict even more reactive vinylazaarene dienophiles, which may be useful in organic synthesis.

## Introduction

It is well known that the Diels–Alder cycloaddition reaction, arguably one of the most useful transformations in organic chemistry,^1,2^ can be greatly accelerated in the presence of catalytic amounts of a Lewis acid (LA).^3^ Typically, the LA binds the dienophile, resulting in a significant stabilization of the lowest unoccupied molecular orbital (LUMO) of the LA-dienophile complex, which is translated into a more favorable highest occupied molecular orbital (HOMO) (diene)–LUMO (dienophile) gap, ultimately leading to the observed acceleration.^4,5^ In addition, the LA-catalyzed Diels–Alder reactions are not only faster than their parent uncatalyzed processes but can also proceed with higher regio- and stereoselectivities.^3^ For instance, recent examples have shown that the inherent *endo*-selectivity of the cycloaddition can be reversed (i.e., favoring the corresponding *exo*-cycloadduct) using sterically overcrowded LA catalysts.^6^

**Scheme 1:**
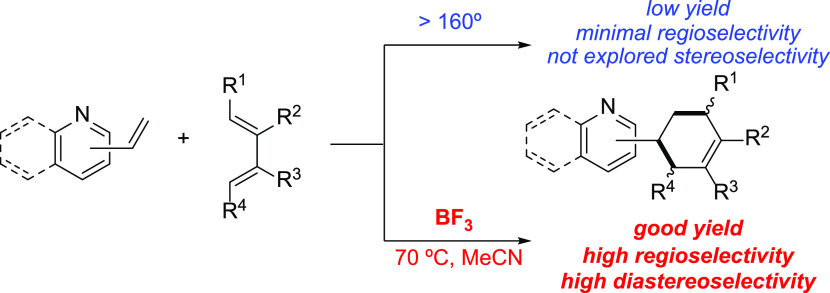
Uncatalyzed and BF_3_-catalyzed Diels–Alder Cycloaddition Reactions Involving Vinylazaarenes and Butadienes

In this regard, Hilinski and co-workers very recently reported^7^ that the highly inefficient and unselective Diels–Alder reaction involving different dienes such as butadiene or isoprene and vinylpyridines^8^ can be transformed into a synthetically useful reaction by simply adding catalytic amounts (0.5 equiv) of the BF_3_ Lewis acid (Scheme 1). The activation of the dienophile via binding of the pyridine lone pair to the LA makes the process not only much faster but also highly regio- and *endo*-diastereoselective, which sharply contrasts with the analogous uncatalyzed cycloadditions.^8^ In addition, this synthetic protocol seems general as it was successfully expanded to a good variety of dienes and different vinylazaarenes, including 2- or 4-vinylpyridines, quinolines, pyrazines, and pyrimidines.^7^

The observed great acceleration of the cycloaddition was rationalized by invoking the above-mentioned traditional LUMO-lowering concept^4,5^ in view of the significant stabilization of the LUMO of the dienophile upon binding to BF_3_.^7^ We have, however, recently demonstrated that this LUMO-lowering concept in slightly related LA-catalyzed Diels–Alder is rather incomplete as it does not consider the impact on the reverse HOMO (dienophile)–LUMO (diene) interaction, which indeed can offset the favorable HOMO (diene)–LUMO (dienophile) interaction.^9^ As a result, we found that the reduction of the Pauli repulsion between the key occupied π-molecular orbitals and not the above orbital interactions constitutes the actual physical mechanism behind the acceleration promoted by LAs in Diels–Alder reactions. This so-called Pauli-repulsion lowering concept^10^ seems general as it applies also in related cycloadditions where the catalyst establishes noncovalent interactions (hydrogen,^11^ halogen,^12^ or chalcogen bonds^13^) with the dienophile and even in slightly related catalyzed Michael-addition reactions^14^ and iminium-catalyzed cycloadditions.^15^ Therefore, we hypothesized that the Pauli-repulsion lowering and not the proposed LUMO-lowering arguments would constitute the actual factor governing the catalysis in this particular BF_3_-mediated cycloaddition reaction involving vinylazaarenes. To check this, we will apply the combination of the activation strain model (ASM)^16^ of reactivity with the energy decomposition analysis (EDA)^17^ method, which was proven to provide detailed quantitative insight into the ultimate factors controlling fundamental processes in organic, main group and organometallic chemistry.^18^ In addition, we shall also apply the ASM-EDA approach to rationalize the reasons behind the almost complete regio- and diastereoselectivity observed in the transformation, which remains completely unknown so far.

## Theoretical Methods

### Activation Strain Model of Reactivity and Energy Decomposition Analysis

Within the ASM method,^16^ also known as the distortion/interaction model,^16b^ the potential energy surface Δ*E*(ζ) is decomposed along the reaction coordinate, ζ, into two contributions, namely the strain Δ*E*_strain_(ζ) associated with the deformation (or distortion) required by the individual reactants during the process and the interaction Δ*E*_int_(ζ) between these increasingly deformed reactants
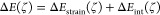
Within the energy decomposition analysis (EDA) method,^17^ the interaction energy can be further decomposed into the following chemically meaningful terms
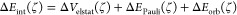
The term Δ*V*_elstat_ corresponds to the classical electrostatic interaction between the unperturbed charge distributions of the deformed reactants and is usually attractive. The Pauli repulsion Δ*E*_Pauli_ comprises the destabilizing interactions between occupied orbitals and is responsible for any steric repulsion. The orbital interaction Δ*E*_orb_ accounts for bond pair formation, charge transfer (interaction between occupied orbitals on one moiety with unoccupied orbitals on the other, including HOMO–LUMO interactions), and polarization (empty-occupied orbital mixing on one fragment due to the presence of another fragment). Moreover, the natural orbital for chemical valence (NOCV)^19^ extension of the EDA method has also been used to further partition the Δ*E*_orb_ term. The EDA-NOCV approach provides pairwise energy contributions for each pair of interacting orbitals to the total bond energy.

## Results and Discussion

**Figure 1:**
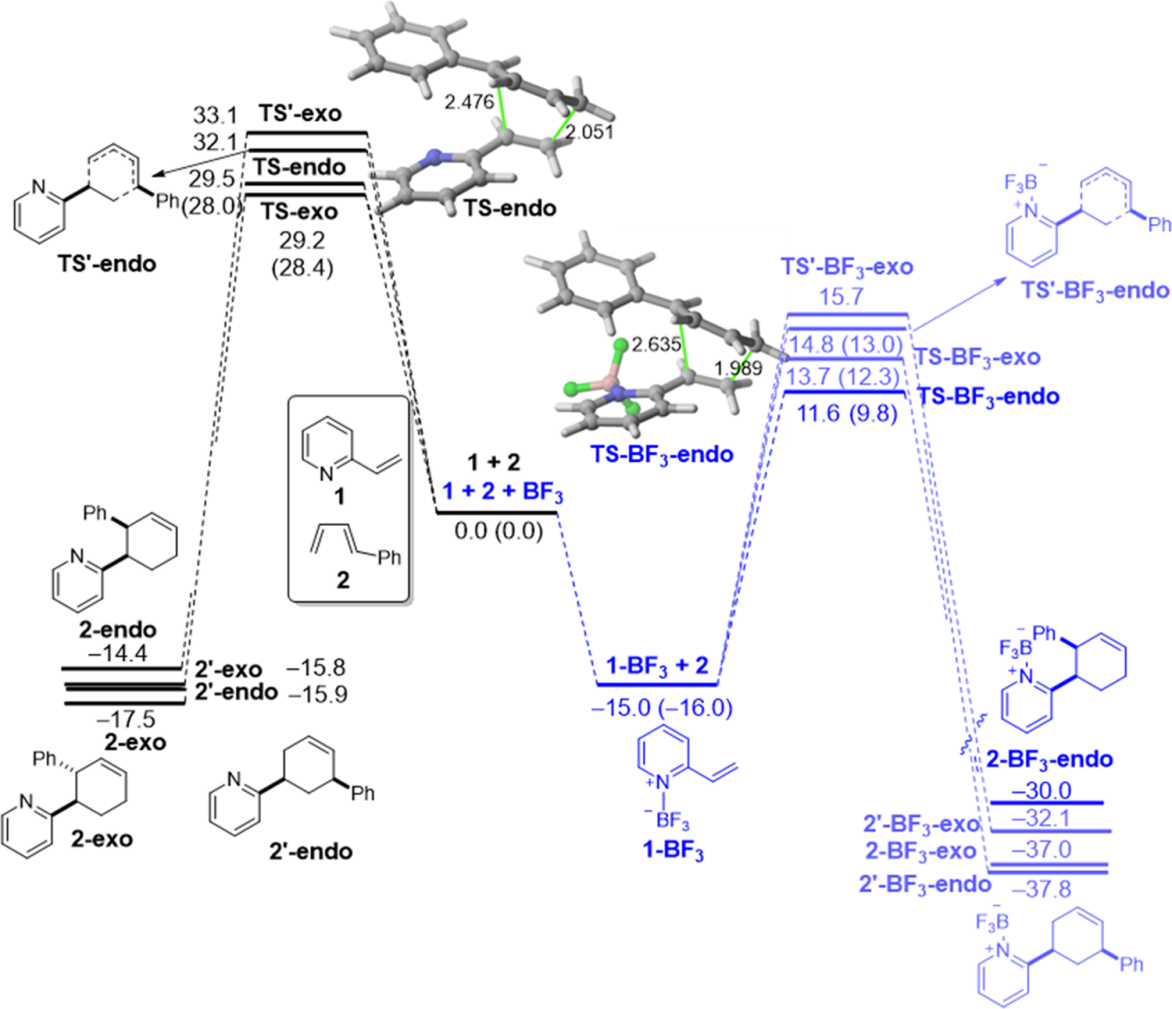
Computed reaction profiles for the uncatalyzed (black) and BF_3_-catalyzed (blue) Diels–Alder cycloaddition reactions involving 2-vinylpyridine (**1**) and 1-phenyl-1,3-butadiene (**2**). Relative Gibbs free energies (in kcal/mol, at 298 K) were computed at the PCM(acetonitrile)-M06-2X/def2-TZVP level. Values within parentheses refer to relative free energies computed at the CPCM(acetonitrile)-DLPNO-CCSD(T)/def2-TZVP//PCM(acetonitrile)-M06-2X/def2-TZVP level.

We first compared the parent uncatalyzed reaction involving 2-vinylpyridine (**1**) and *trans*-1-phenyl-1,3-butadiene (**2**) with the analogous cycloaddition reaction mediated by BF_3_. Our calculations (PCM(acetonitrile)-M06-2X/def2-TZVP level) indicate that, in both cases, the transformation proceeds concertedly through the corresponding asynchronous, six-membered transition state, which leads to the exergonic formation of the respective cycloadduct (see Figure 1). As expected, the BF_3_-catalyzed reaction involves the initial activation of the dienophile, thus forming the donor-acceptor complex **1-BF**_**3**_ in a highly exergonic reaction (Δ*G*_R_ = −15.0 kcal/mol). From the data in Figures 1 and S1 (the latter showing the reaction profiles computed at 70 °C), it becomes evident that this activation renders the BF_3_-mediated process much more favored than the uncatalyzed reaction along the entire reaction coordinate. In particular, the reduction in the cycloaddition barrier (ΔΔ*G*^≠^ = 2.9 kcal/mol and 3.4 kcal/mol, computed at 25 and 70 °C, respectively, for the *endo*-pathway) is consistent with the acceleration induced by the BF_3_ catalyst observed experimentally.^7^ In addition, the high activation barrier computed for the uncatalyzed reaction (Δ*G*^≠^ ≈ 32 kcal/mol, at 70 °C) is also consistent with the low yield observed experimentally (ca. 3%, at 70 °C). Moreover, the rather low energy for the isosdemic reaction **1** + **2-BF**_**3**_**-endo** → **1-BF**_**3**_ + **2-endo** (Δ*G* = 0.6 kcal/mol, either at 25 or 70 °C) indicates a high degree of completion of the catalytic cycle.

Our calculations also reproduce both the almost complete regio- and diastereoselectivity observed experimentally.^7^ As shown in Figure 1, the *endo*-cycloadduct **2-BF**_**3**_**-endo** is preferentially formed under kinetic control in view of the higher barrier computed for the formation of the corresponding *exo*-cycloadduct (ΔΔ*G*^≠^ = 2.1 kcal/mol), which is consistent with the experimental diasteromeric ratio of >20:1. Similarly, the almost complete regioselectivity (>20:1) also occurs under kinetic control as the barrier computed for the formation of the alternative cycloadduct **2′-BF**_**3**_**-endo** is 3.2 kcal/mol higher than that computed for the major isomer **2-BF**_**3**_**-endo**. Rather similar activation barriers were computed at the highly accurate CPCM(acetonitrile)-DLPNO-CCSD(T)/def2-TZVP level (see Figure 1), which provides further support to the chosen computational level for this study.

**Figure 2:**
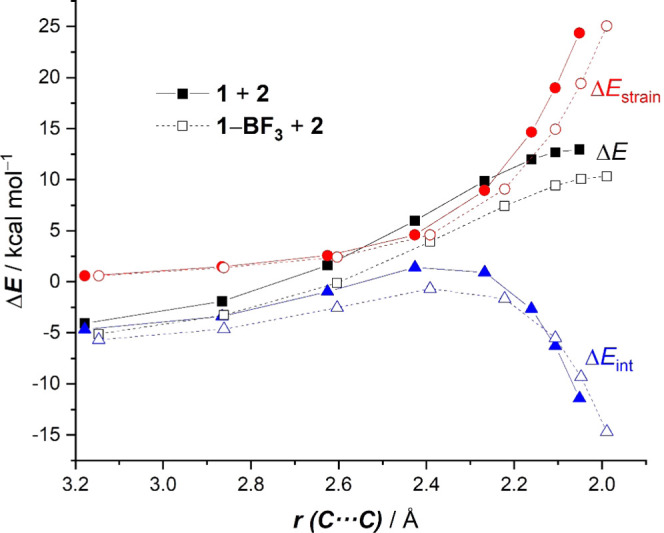
Comparative activation strain analyses of the Diels–Alder cycloaddition reactions between 1-phenyl-butadiene (**2**) and 2-vinylpyridine (**1**) (uncatalyzed, solid lines) and the 2-vinylpyridine-BF_3_ complex (**1-BF**_**3**_) (dotted lines) projected onto the shorter C···C bond-forming distance. All data have been computed at the PCM(acetonitrile)-M06-2X/def2-TZVP level.

To understand the reasons behind the computed acceleration of the BF_3_-mediated process, the activation strain model was applied next. To enable a direct comparison, we focused on the uncatalyzed and catalyzed cycloadditions leading to the corresponding *endo*-cycloadducts. Figure 2 shows the computed activation strain diagrams (ASDs) for both reactions from the initial stages of the transformation to the respective transition states and projected onto the shorter C···C bond-forming distances.^20^ From the data in Figure 2, it becomes clear that the BF_3_-mediated reaction benefits from both a less destabilizing strain energy (measured by the Δ*E*_strain_ term) and a stronger interaction between the deformed reactants (measured by the Δ*E*_int_ term) along practically the entire reaction coordinate and particularly at the transition state region. We can ascribe the trend in Δ*E*_strain_ to the extent of the asynchronicity of the cycloaddition, which is markedly higher in the BF_3_-reaction (uncatalyzed: Δ*r*_C···C_^TS^ = 0.425 Å < catalyzed: Δ*r*_C···C_^TS^ = 0.646 Å, where Δ*r*_C···C_^TS^ is the difference between the newly forming C···C bond lengths in the TS, see Figure 1). Therefore, a higher asynchronicity value implies that the corresponding transition state is reached earlier, and consequently, the energy penalty to adopt the TS-geometry is lower.

**Figure 3:**
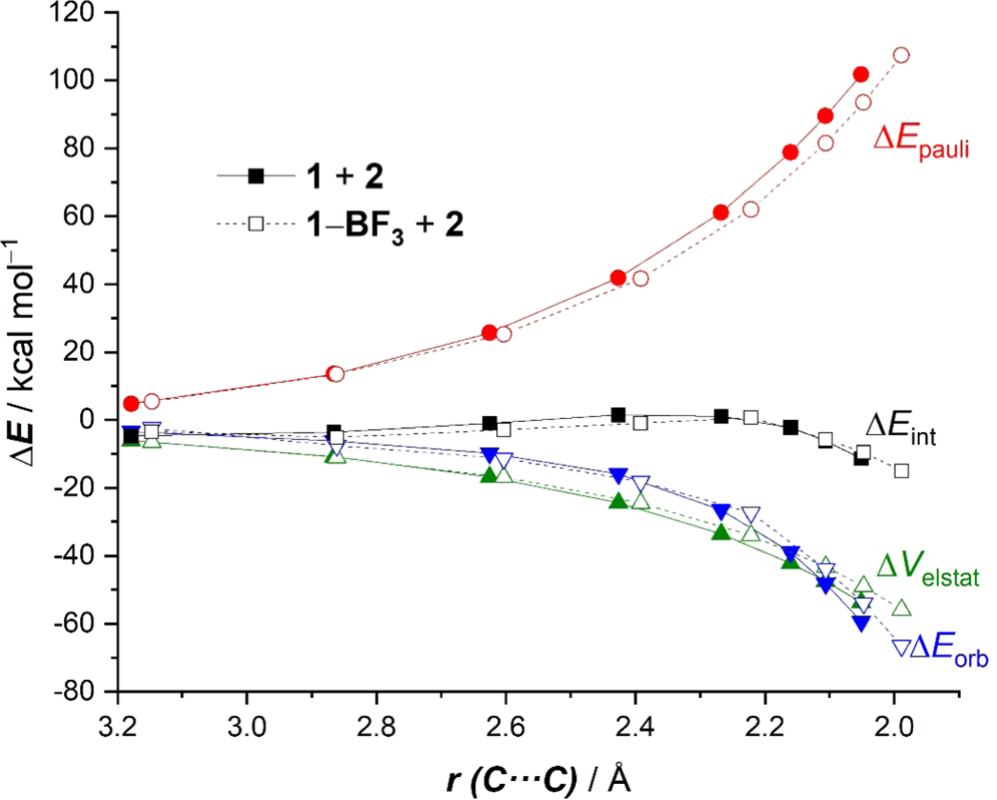
Comparative energy decomposition analyses of the Diels–Alder cycloaddition reactions between 1-phenyl-butadiene (**2**) and 2-vinylpyridine (**1**) (uncatalyzed, solid lines) and the 2-vinylpyridine-BF_3_ complex (**1-BF**_**3**_) (dotted lines). All data have been computed at the ZORA-M06-2X/TZ2P//PCM(acetonitrile)-M06-2X/def2-TZVP level.

The origin of the above-mentioned stronger interaction between the deformed reactants computed for the BF_3_-mediated cycloaddition can be found with the help of the energy decomposition analysis. As shown in Figure 3, which graphically shows the evolution of the EDA terms along the reaction coordinate for both the uncatalyzed and BF_3_-catalyzed cycloadditions, it becomes clear that both attractive (electrostatic, Δ*V*_elstat_, and orbital, Δ*E*_orb_) interactions are slightly more stabilizing for the uncatalyzed reaction than for the BF_3_-cycloaddition. For instance, at the same consistent C···C bond-forming distance of 2.1 Å,^21^ the difference in both terms is ΔΔ*V*_elstat_ = 4.3 kcal/mol and ΔΔ*E*_orb_ = 4.4 kcal/mol, favoring the uncatalyzed reaction, which indicates that neither the electrostatic attractions nor the orbital interactions (despite the more favorable HOMO (diene)–LUMO (dienophile) gap) are responsible for the higher interaction computed for the BF_3_-catalyzed reaction. At variance, data in Figure 3 clearly suggest that the catalyzed process benefits from a less destabilizing Pauli repulsion between occupied orbitals (mainly the π-HOMO-2(diene)−π-HOMO(dienophile) interaction) practically along the entire reaction coordinate. The lower Δ*E*_Pauli_ value computed for the BF_3_-mediated cycloaddition results from the polarization induced by the Lewis acid of the occupied π-molecular orbital on the reactive C=C bond of the dienophile, as confirmed by the decrease in the natural charge of the reactive terminal C=CH_2_ carbon atom (−0.360e in **1** vs −0.317e in **1-BF**_**3**_). Therefore, this Pauli-repulsion lowering effect and not the proposed LUMO-lowering^7^ (together with the computed lower strain energy) is the ultimate factor responsible for the lower barrier of the BF_3_-mediated cycloaddition reaction.

### Endo/Exo Selectivity

Once we have disclosed the factors controlling the catalysis in this cycloaddition, we then focus on those factors responsible for the remarkable *endo/exo* selectivity (>20:1) observed experimentally.^7^ From the data in Figure 1, the remarkable influence of the Lewis acid on the diastereoselectivity of the process becomes evident. Whereas almost no selectivity is found for the parent uncatalyzed reaction (ΔΔ*G*^≠^ = 0.3 kcal/mol favoring the *exo*-cycloadduct), a clear *endo*-preference (ΔΔ*G*^≠^ = 2.1 kcal/mol) is computed for the BF_3_-mediated cycloaddition. The latter barrier energy difference is slightly reduced to ΔΔ*G*^≠^ = 2.0 kcal/mol when computed at 343 K (the temperature used in the experiments), which is translated into a 23:1 selectivity, therefore nearly matching the observed *endo*/*exo* ratio.

**Figure 4:**
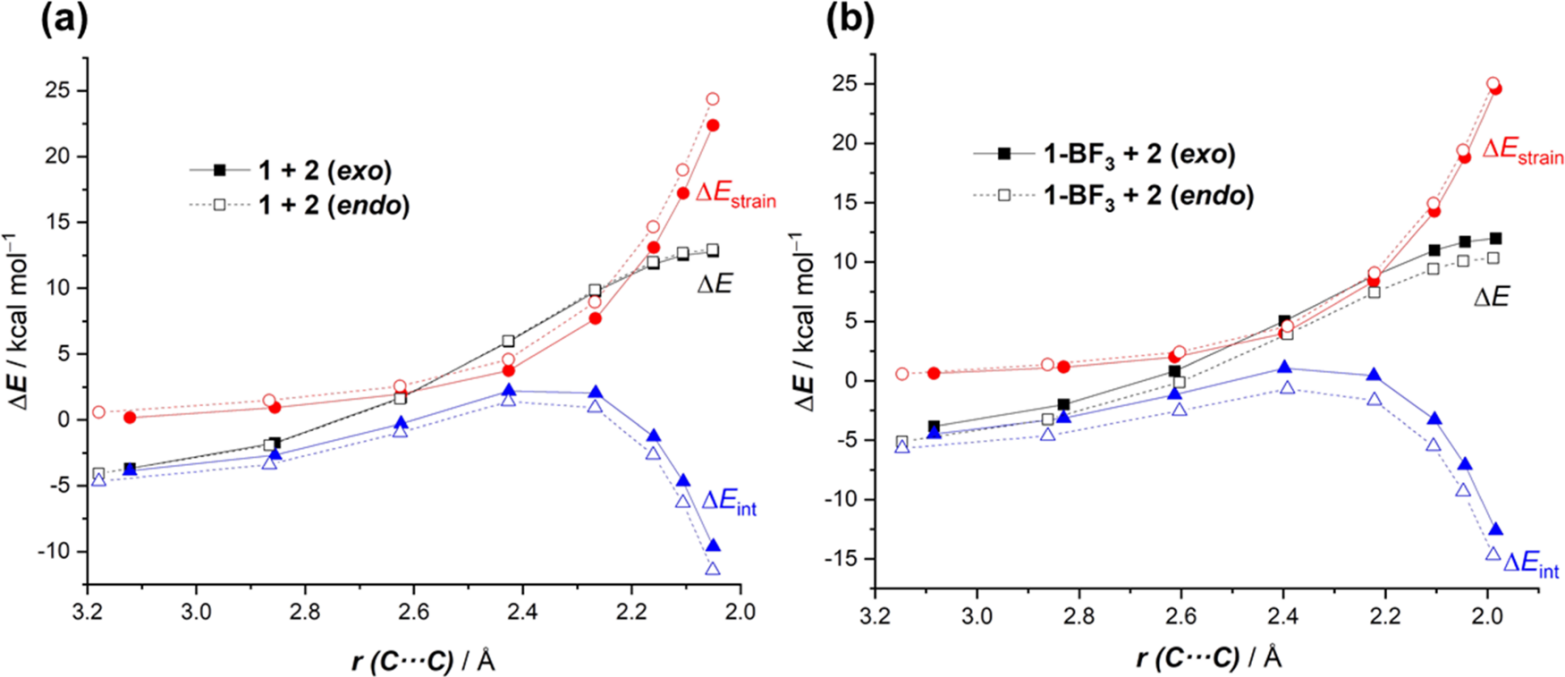
Comparative activation strain analyses of the Diels–Alder cycloaddition reactions between (a) 1-phenyl-butadiene (**2**) and 2-vinylpyridine (**1**) and (b) 1-phenyl-butadiene (**2**) and the 2-vinylpyridine-BF_3_ complex (**1-BF**_**3**_) for the *endo* (dotted lines) and *exo* (solid lines) pathways projected onto the shorter C···C bond-forming distance. All data have been computed at the PCM(acetonitrile)-M06-2X/def2-TZVP level.

The ASM was applied again to quantitatively understand this markedly different selectivity in the presence of BF_3_. From the data in Figure 4a, which shows the corresponding ASDs for the uncatalyzed reaction, it can be seen that the *exo*-approach benefits from a less destabilizing strain energy. However, the interaction between the deformed reactants is clearly more stabilizing for the *endo*-pathway along the entire reaction coordinate, which offsets the Δ*E*_strain_ term, therefore resulting in nearly identical barriers for both approaches. Similarly, for the BF_3_-mediated process, the *endo*-pathway benefits from a stronger interaction between the deformed reactants, but at variance with the uncatalyzed reaction, the strain energy becomes rather similar for both approaches (Figure 4b). As a consequence, the *endo*-pathway becomes more stabilized and kinetically preferred over the *exo*-path. This behavior is also different from that found for the parent reaction between cyclopentadiene and maleic anhydride where the *endo*-selectivity is derived exclusively from the strain energy^22^ but strongly resembles that in related cycloaddition reactions mediated by bidentate bis-selenonium cations, which also act as Lewis acid catalysts.^13^ According to the EDA method (see Figure S2), the stronger Δ*E*_int_ computed for the *endo*-pathway is mainly the result of stronger electrostatic and orbital (albeit to a lesser extent) interactions and not of the Pauli repulsion, which is slightly less destabilizing for the *exo*-pathway. According to the NOCV extension of the EDA method, the stronger orbital interactions computed for the *endo*-pathway mainly result from a higher reverse π-LUMO(diene) ← π-HOMO(dienophile), particularly, at the proximities of the transition state.

### Regioselectivity

**Figure 5:**
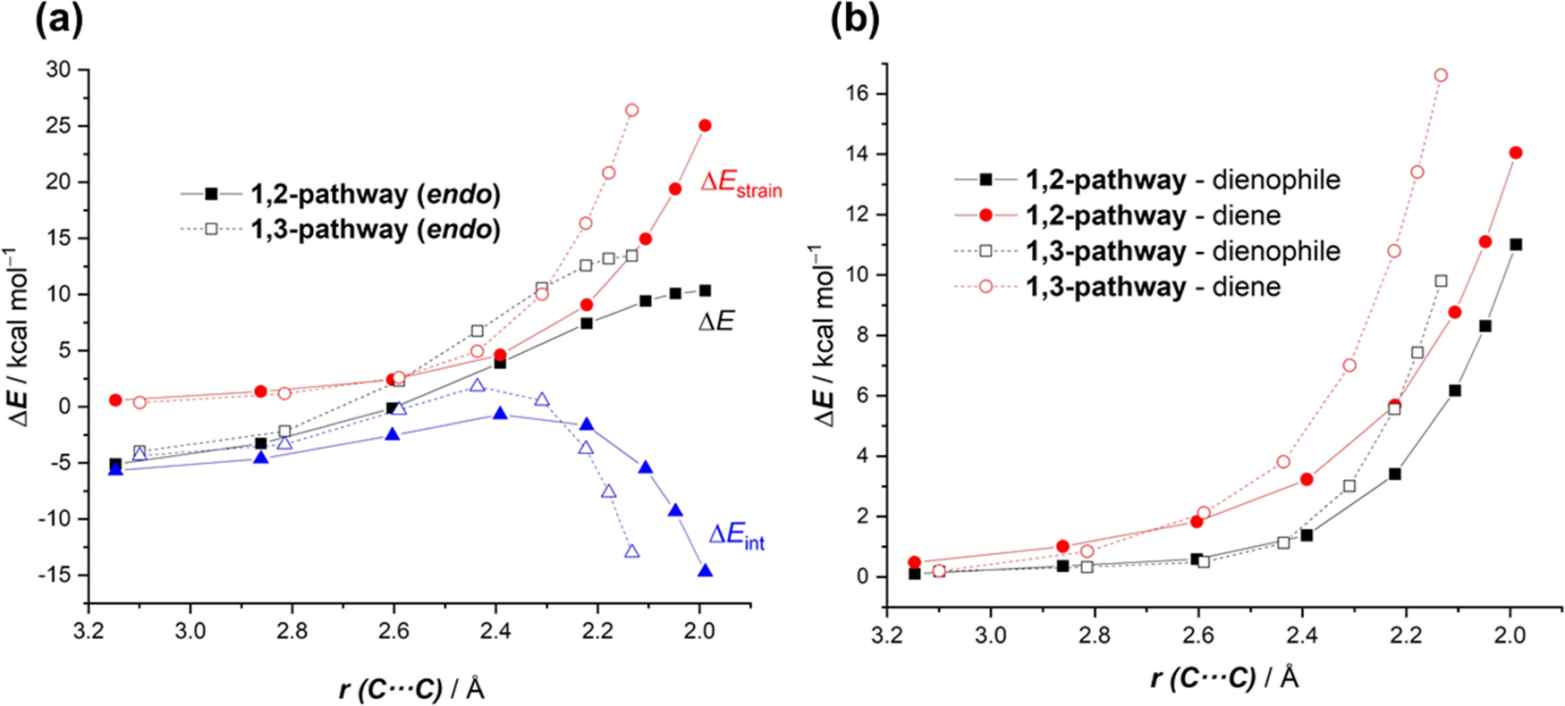
(a) Comparative activation strain diagrams for the cycloaddition reactions between 1-phenyl-butadiene (**2**) and 2-vinylpyridine-BF_3_ complex (**1-BF**_**3**_) (dotted lines) for the competitive 1,2-pathway (solid lines) and 1,3-pathway (dotted lines) projected onto the shorter C···C bond-forming distance. (b) Decomposition of the total strain into contributions coming from each reactant. All data have been computed at the PCM(acetonitrile)-M06-2X/def2-TZVP level.

Data in Figure 1 also indicate that the cycloaddition reaction involving **1-BF**_**3**_ and **2** is completely selective toward the formation of the 1,2-cycloadduct **2-BF**_**3**_**-endo** at the expense of the corresponding 1,3-cycloadduct **2′-BF**_**3**_**-endo** (ΔΔ*G*^≠^ = 3.2 kcal/mol), which is again consistent with the experimental findings.^7^ According to the ASM method, the higher barrier of the 1,3-pathway derives almost exclusively from a more destabilizing strain energy as compared to the favored 1,2-pathway, which in addition benefits from a stronger interaction at the transition state structure (Figure 5a). The partitioning of the key Δ*E*_strain_ term into contributions coming from both reactants (Figure 5b) indicates that the higher (i.e., more destabilizing) total strain computed for the 1,3-pathway originates from the higher distortion required by both the dienophile and the diene (albeit to a lesser extent) reactants to adopt the geometry of the saddle point **TS′-BF**_**3**_**-endo** in comparison to the more stable **TS-BF**_**3**_**-endo**.

### Extension to 4-Vinylpyrinide and Related Compounds

**Figure 6:**
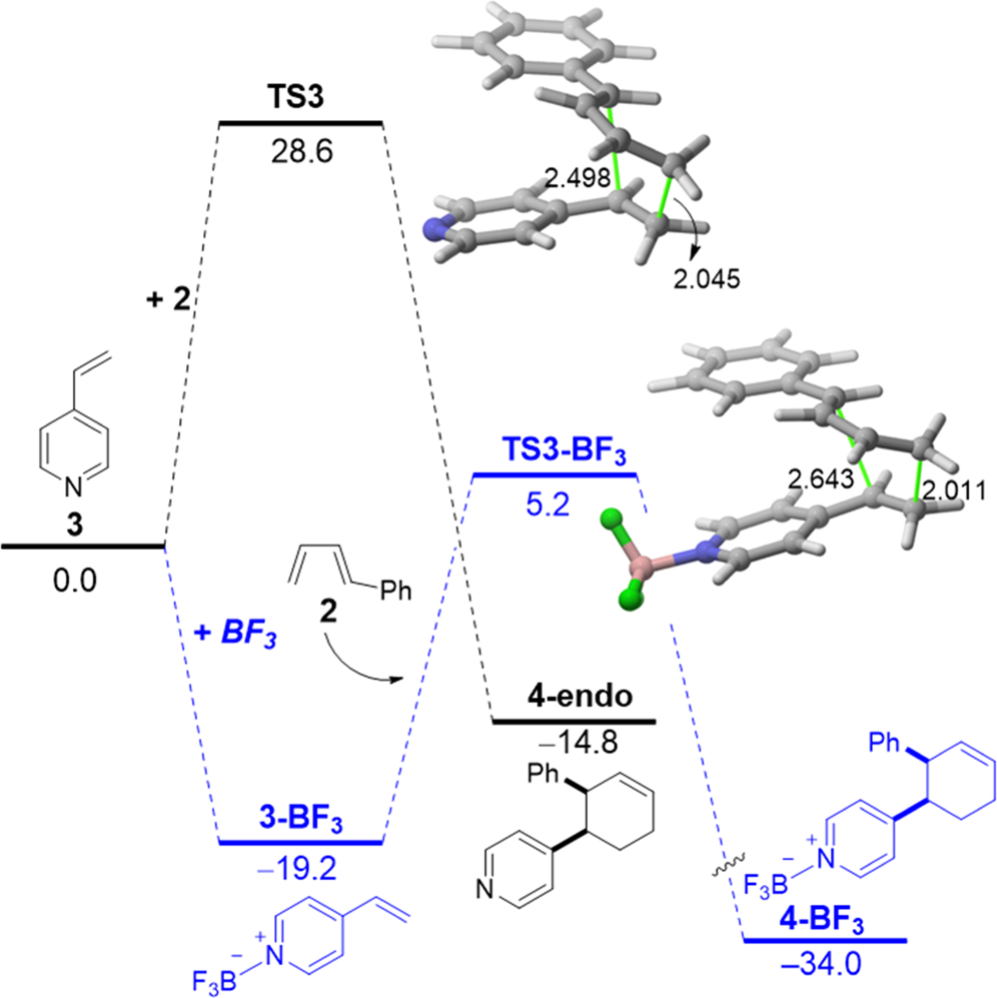
Computed reaction profiles for the uncatalyzed (black) and BF_3_-catalyzed (blue) Diels–Alder cycloaddition reactions involving 4-vinylpyridine (**3**) and 1-phenyl-1,3-butadiene (**2**). Relative Gibbs free energies (in kcal/mol, at 298 K) were computed at the PCM(acetonitrile)-M06-2X/def2-TZVP level.

The available experimental data indicate that a similar reactivity enhancement promoted by BF_3_ is found when using related vinylazaarenes such as 4-vinylpyridines, pyrimidines, or quinolines.^7^ Our calculations are in line with this and confirm that the cycloaddition involving the same diene (*trans*-1-phenyl-1,3-butadiene, **2**) and 4-vinylpyridine (**3**) (only the preferred *endo*-pathway is considered, see Figure 6) becomes much more favored in the presence of BF_3_ along the entire reaction coordinate. In comparison with the analogous process involving 2-vinylpyridine (**1**, see Figure 1), the transformation involving 4-vinyplyridine is even more favored along the entire process, from the initial Lewis acid complex **3-BF**_**3**_ to the final cycloadduct **4-BF**_**3**_. This suggests that the polarization induced by the catalysis is even more effective when the reactive alkene and the N-BF_3_ moiety are placed in a 1,4-relative position rather than in a 1,2-relative position, which is supported by the lower natural charge of the reactive terminal C=CH_2_ carbon atom (−0.304e vs −0.317e in **3-BF**_**3**_ and **1-BF**_**3**_, respectively).

**Figure 7:**
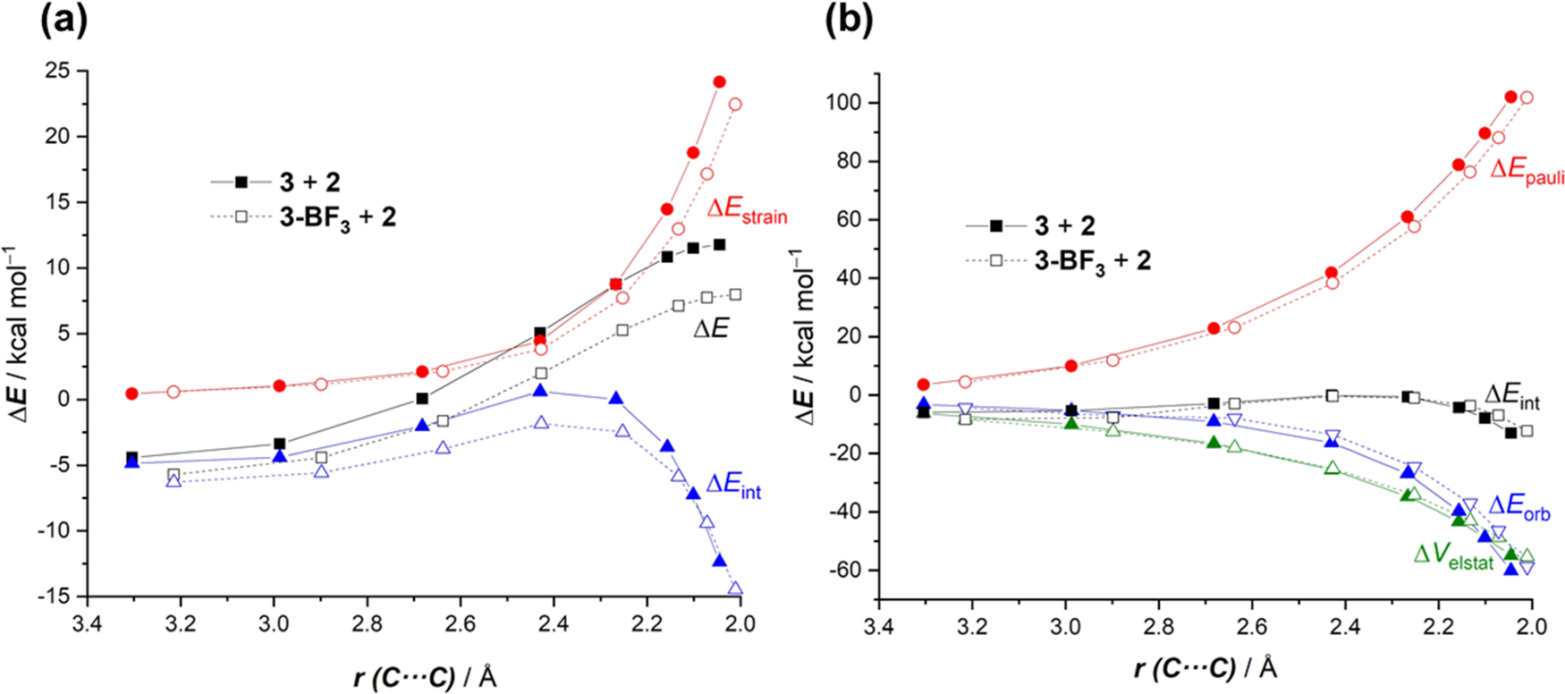
Comparative activation strain analyses (a) PCM(acetonitrile)-M06-2X/def2-TZVP level and energy decomposition analysis (b) ZORA-M06-2X/TZ2P//PCM(acetonitrile)-M06-2X/def2-TZVP level of the Diels–Alder cycloaddition reactions between 1-phenyl-butadiene (**2**) and 2-vinylpyridine (**3**) (solid lines) and the 2-vinylpyridine-BF_3_ complex (**3-BF**_**3**_) (dotted lines) projected onto the shorter C···C bond-forming distance.

The above-mentioned depopulation of the reactive alkene moiety induced by the Lewis acid points again to the Pauli-repulsion lowering as a critical factor controlling the cycloaddition involving 4-vinylpyridine. To confirm this, we applied the combination of the ASM and EDA methods. Once again, it is shown that the BF_3_-catalyzed reaction benefits from a less stabilizing strain energy together with a stronger interaction between the deformed reactants along the entire reaction coordinate (Figure 7a). The trend in the Δ*E*_strain_ term can be again ascribed to the higher asynchronicity of the BF_3_-mediated process (uncatalyzed: Δ*r*_C···C_^TS^ = 0.453 Å < catalyzed: Δ*r*_C···C_^TS^ = 0.632 Å), whereas the stronger Δ*E*_int_ term results, according to the EDA method (Figure 7b), exclusively from a reduced Pauli repulsion (Δ*E*_Pauli_). Therefore, it is confirmed that the Lewis acid acts as an electron-withdrawing group, which depopulates the reactive π-C=C molecular orbital of the dienophile reducing the Pauli repulsion with the diene and making the process more asynchronous. Both effects, and not the previously proposed more favorable HOMO (diene)–LUMO (dienophile) orbital interaction,^7^ constitute therefore the ultimate factors leading to the observed acceleration of this cycloaddition reaction.

**Figure 8:**
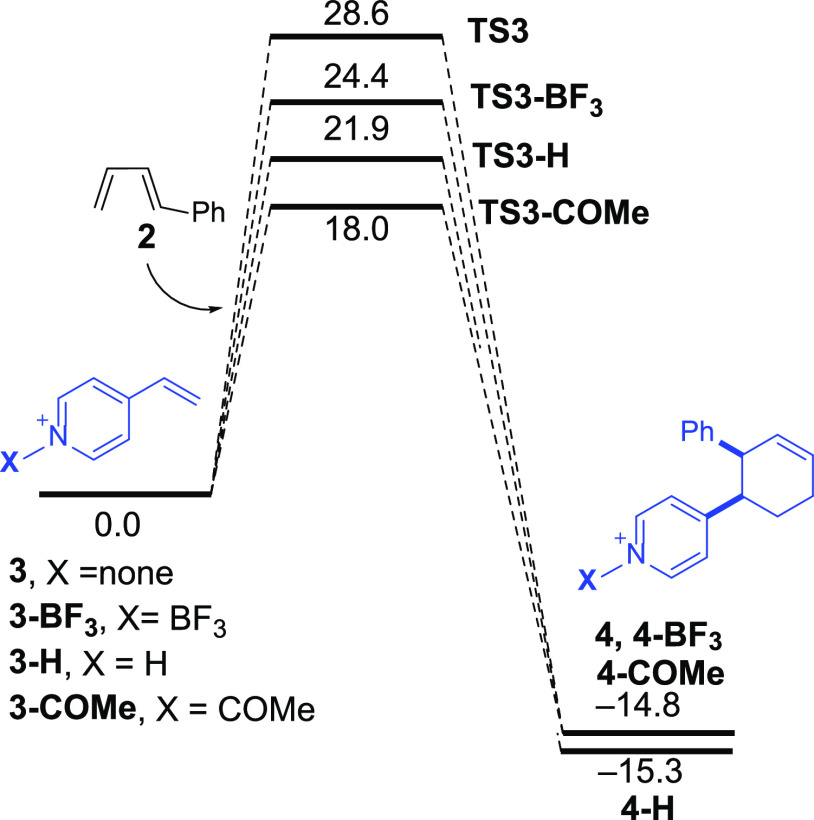
Computed reaction profiles for the Diels–Alder cycloaddition reactions involving 4-vinylpyridines (**3**) and 1-phenyl-1,3-butadiene (**2**). Relative Gibbs free energies (in kcal/mol, at 298 K) were computed at the PCM(acetonitrile)-M06-2X/def2-TZVP level.

The above results suggest that the activation barrier of the cycloaddition involving vinylpyridines as dienophiles could be further reduced by increasing the acceptor ability of the pyridine nitrogen atom. This may be achieved simply by protonation (**3-H**) or acetylation (**3-COMe**; see Figure 8). Indeed, our calculations indicate that the depopulation of the key π-C=C molecular orbital is even greater in these cationic dienophiles (natural charge of the terminal carbon atom of −0.279e and −0.259e, respectively), and for this reason, it is not surprising that lower activation barriers were computed for the analogous cycloaddition reactions involving these positively charged species (Figure 8).

**Figure 9:**
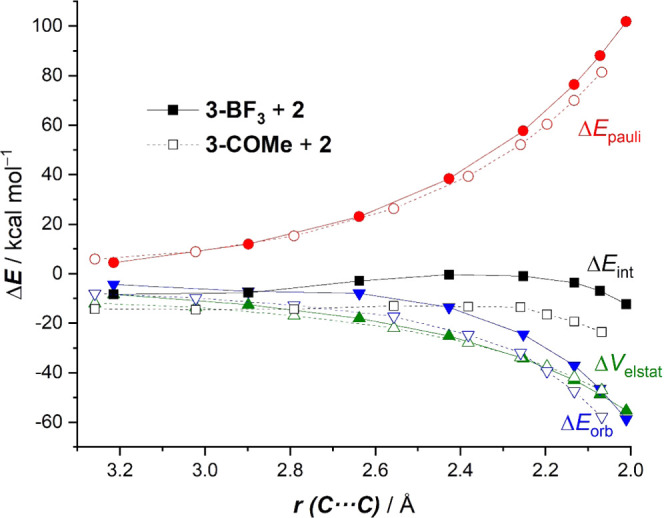
Comparative energy decomposition analyses of the Diels–Alder cycloaddition reactions between 1-phenyl-butadiene (**2**) and 4-vinylpyridines **3-BF**_**3**_ (solid lines) and **3-COMe** (dotted lines). All data have been computed at the ZORA-M06-2X/TZ2P//PCM(acetonitrile)-M06-2X/def2-TZVP level.

Considering the above results, one might initially ascribe the increased reactivity of **3-H** or **3-COMe** with respect to **3-BF**_**3**_ to a further reduction of the Pauli repulsion between the key occupied π-orbitals of the diene and dienophile, and indeed, this is confirmed by the EDA method (see Figure 9 for the analyses of the representative reactions involving **3-BF**_**3**_ and **3-COMe**). However, from the evolution of the EDA terms in Figure 9, it becomes clear that the reduction of the Pauli repulsion is, in this particular case, not the only factor leading to the more stabilizing interaction between the deformed reactants in the **3-COMe** + **2** cycloaddition reaction. In addition, the process involving this cationic dienophile also benefits from much stronger orbital interactions along the entire reaction coordinate. In fact, the enhancement of the Δ*E*_orb_ interactions in the process involving **3-COMe** is even more pronounced than the reduction in the Pauli repulsion. For instance, at the same consistent C···C bond-forming distance of 2.1 Å, ΔΔ*E*_orb_ = 11.1 kcal/mol, whereas a lower value was computed for the difference in the Pauli repulsion, ΔΔ*E*_Pauli_ = −6.8 kcal/mol. This is markedly different from the process involving **3-BF**_**3**_ in comparison with the uncatalyzed reaction involving **3** (Figure 7), where the orbital interactions are more stabilizing for the latter reaction (see above). Therefore, it can be concluded that the further acceleration computed for the cycloadditions involving the cationic dienophiles **3-H** or **3-COMe** finds its origin not only in a reduction of the Pauli repulsion, as it occurs in the analogous reactions involving **BF**_**3**_-complexed vinylpyridines, but also in a remarkable enhancement of the orbital interactions between the deformed reactants.

**Figure 10:**
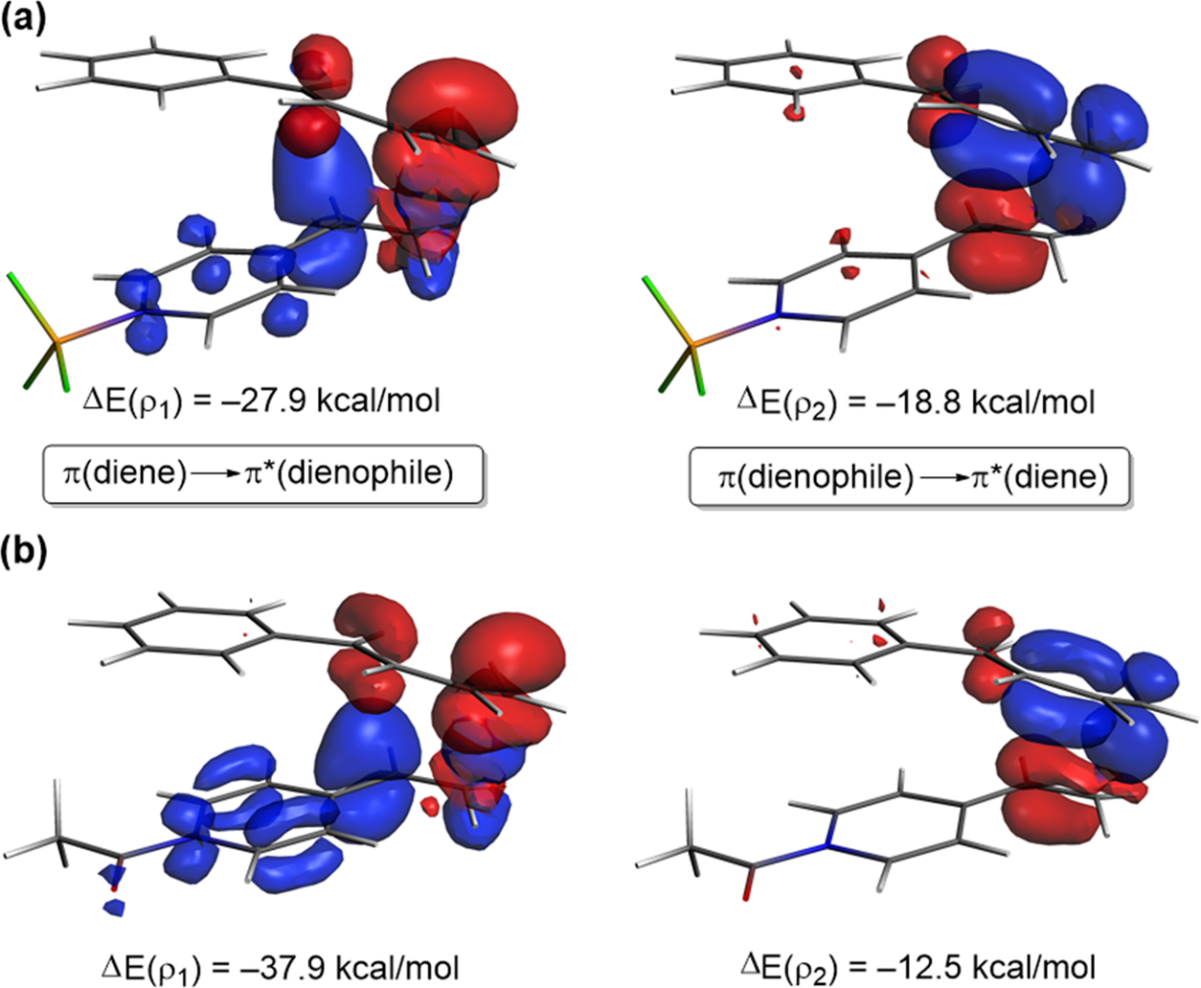
Plot of the deformation densities Δρ of the pairwise orbital interactions between the interacting fragments and the corresponding stabilization energies Δ*E*(ρ) computed for the Diels–Alder cycloaddition reactions between 1-phenyl-butadiene (**2**) and 4-vinylpyridines **3-BF**_**3**_ (a) and **3-COMe** (b). The color code of the charge flow is red → blue.

To understand the reasons behind the above-mentioned stronger orbital interactions in the processes involving the cationic dienophiles **3-H** or **3-COMe**, we finally applied the natural orbital for chemical valence (NOCV) extension of the EDA method. Within this approach, we are able to not only identify but also quantify the main orbital interactions contributing to the total Δ*E*_orb_ term. The NOCV method identifies two main orbital interactions, namely the direct π-HOMO(diene) → π*-LUMO(dienophile) interaction and the reverse π-HOMO(dienophile) → π*-LUMO(diene) interaction, denoted as ρ_1_ and ρ_2_, respectively (see Figure 10). Not surprisingly, our calculations indicate that in both processes the strength of the former interaction is higher than that of the latter (ρ_1_ > ρ_2_), which confirms the normal electron-demand nature of the considered cycloaddition reactions. Interestingly, although the reverse interaction ρ_2_ is weaker in the process involving the cationic dienophile (ΔΔ*E*(ρ_2_) = −6.3 kcal/mol), the key direct orbital interaction ρ_1_ is significantly increased (ΔΔ*E*(ρ_1_) = 10.0 kcal/mol), which results in the higher orbital interactions (and lower barrier) computed for this reaction.

## Conclusions

The present computational study provides detailed quantitative insight into the factors controlling the Lewis acid-catalyzed Diels–Alder cycloaddition reaction involving vinylazaarenes. It is found that, in comparison with the parent uncatalyzed reaction, the BF_3_-promoted cycloaddition is greatly accelerated not because of the stabilization of the LUMO of the dienophile but to a significant reduction of the Pauli repulsion between the deformed reactants together with the higher asynchronicity of the corresponding transition states. In addition, the process is highly *endo*-selective and produces almost exclusively the corresponding 1,2-cycloadduct. While the *endo*-selectivity can be mainly ascribed to stronger electrostatic and orbital interactions between the deformed reactants in the *endo*-approach, the 1,2-pathway benefits from a less destabilizing strain in comparison with the alternative 1,3-pathway. Our results indicate that the Lewis acid catalyst provokes a significant depopulation of the reactive π-molecular orbital of the dienophile, which can be even further increased in related cationic systems. In these cases, a significant reactivity enhancement is predicted, which may be useful for synthetic chemists working on cycloaddition reactions involving otherwise low reactive vinylazaarenes.

## Experimental Section

### Computational Details

Geometry optimizations of the molecules were performed without symmetry constraints using the Gaussian-09 (RevD.01)^23^ suite of programs and the hybrid meta-GGA M06-2X functional^24^ in conjunction with the triple-ζ basis set def2-TZVP.^25^ This level of theory has been proven to provide accurate results for organic chemistry reactions.^26^ Solvent effects (solvent = benzene) were taken into account with the polarization continuum model (PCM) method.^27^ This level is denoted as PCM(acetonitrile)-M06-2X/def2-TZVP. Reactants and adducts were characterized by frequency calculations and have positive definite Hessian matrices. Transition states (TSs) show only one negative eigenvalue in their diagonalized force constant matrices, and their associated eigenvectors were confirmed to correspond to the motion along the reaction coordinate under consideration using the intrinsic reaction coordinate (IRC) method.^28^ Additionally, single-point energy refinements were carried out at a highly accurate CPCM(acetonitrile)-DLPNO-CCSD(T)^29^/def2-TZVP//PCM(acetonitrile)-M06-2X/def2-TZVP level for selected steps of the transformation to check the reliability of the selected PCM(acetonitrile)-M06-2X/def2-TZVP level.^30^ It was found that the relative energy differences were not significant, which indicated that the selected DFT level was sufficient for the purpose of the present study (see Figure 1).

The program package ADF^31^ was used for EDA calculations using the optimized PCM(acetonitrile)-M06-2X/def2-TZVP geometries at the same DFT level in conjunction with a triple-ζ-quality basis set using uncontracted Slater-type orbitals (STOs) augmented by two sets of polarization functions with a frozen-core approximation for the core electrons.^32^ Auxiliary sets of s, p, d, f, and g STOs were used to fit the molecular densities and to represent the Coulomb and exchange potentials accurately in each SCF cycle.^33^ Scalar relativistic effects were incorporated by applying the zeroth-order regular approximation (ZORA).^34^ This level of theory is denoted as ZORA-M06-2X/TZ2P//PCM(acetonitrile)-M06-2X/def2-TZVP.

## Supporting Information Available

Cartesian coordinates (in Å) and total energies of all of the stationary points discussed in the text (Figures S1 and S2) (PDF)
