# Supplementary material for: Origin of Catalysis and Selectivity in Lewis Acid-Promoted Diels–Alder Reactions Involving Vinylazaarenes as Dienophiles
Source: J Org Chem. 2022 Jul 7;87(14):9307–15. doi: 10.1021/acs.joc.2c01035 (PMC9295156; doi:10.1021/acs.joc.2c01035)
Supplement: Supplementary file 1 — jo2c01035_si_001.pdf [file jo2c01035_si_001.pdf]

# Origin of Catalysis and Selectivity in Lewis Acid-Promoted Diels-Alder Reactions involving Vinylazaarenes as Dienophiles

Susana Portela and Israel Fernández*

S. Portela and I. Fernández

Departamento de Química Orgánica I and Centro de Innovación en Química Avanzada (ORFEO-CINQA)

Facultad de Ciencias Químicas

Universidad Complutense de Madrid, 28040-Madrid, Spain

e-mail: israel@quim.ucm.es

**Contents:**

**1.** Figure S1……………………………………………………………………S1

**2.** Figure S2……………………………………………………………………S2

**3.** Cartesian coordinates and energies…………………………………………....S3

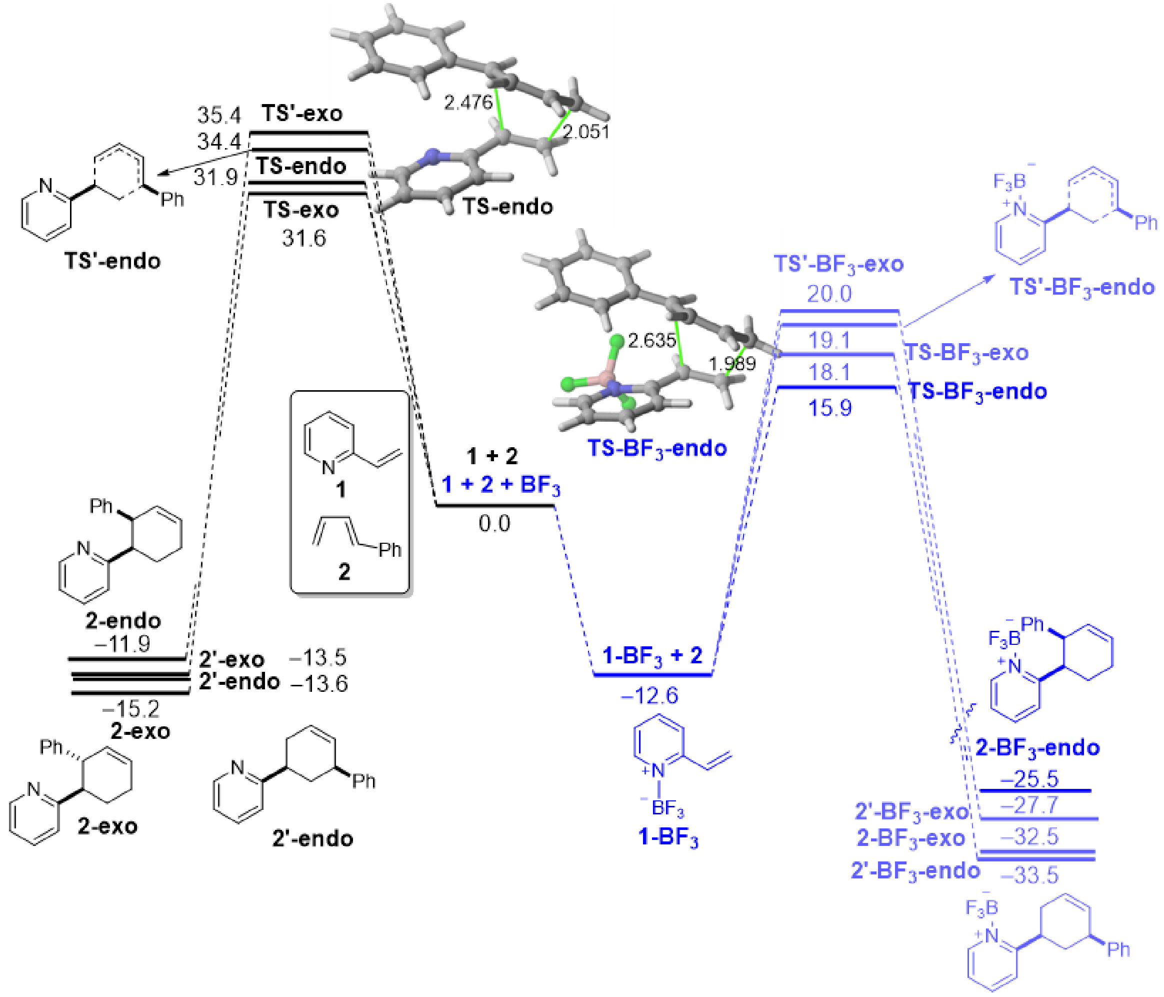


**Figure S1.** Computed reaction profiles for the uncatalyzed (black) and $BF_3$-catalyzed (blue) Diels-Alder cycloaddition reactions involving 2-vinylpyridine (**1**) and 1-phenyl-1,3-butadiene (**2**). Relative Gibbs free energies (in kcal/mol, at 343 K) were computed at the PCM(acetonitrile)-M06-2X/def2-TZVP level.

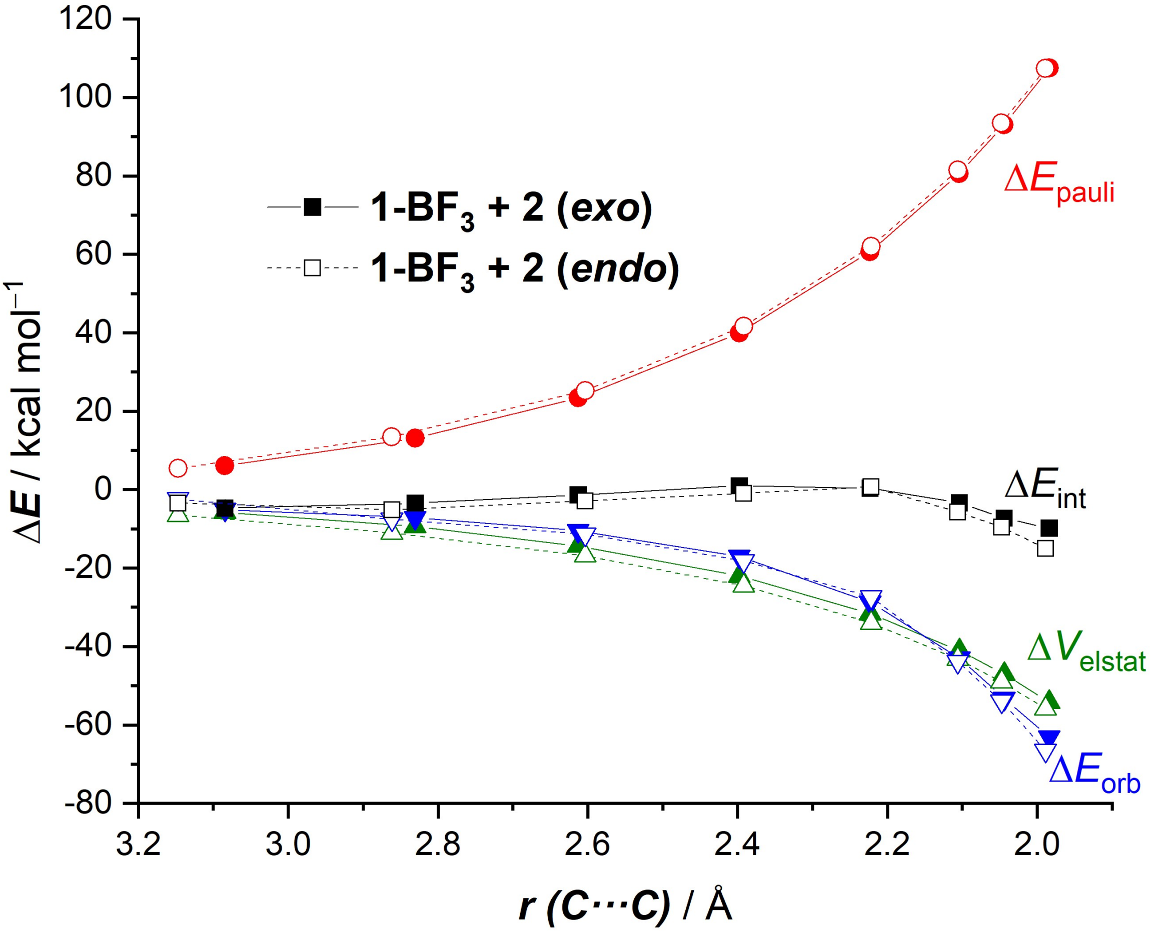


**Figure S2.** Comparative energy decomposition analysis of the Diels-Alder cycloaddition reactions between 1-phenyl-butadiene (**2**) and 2-vinylpyridine-$BF_3$ complex (**1-$BF_3$**) for the *endo* (dotted lines) and *exo* (solid lines) pathways projected onto the shorter C···C bond-forming distance. All data have been computed at the ZORA-M06-2X/TZ2P//PCM(acetonitrile)-M06-2X/def2-TZVP level.

Cartesian coordinates (in Å) and total energies (in a.u., noncorrected ZVPE included) of all the stationary points discussed in the text. All calculations have been performed at the PCM(acetonitrile)-M06-2X/def2-TZVP level. ***SP*** denotes values computed at the CPCM(acetonitrile)-DLPNO-CCSD(T)/def2TZVP//PCM(acetonitrile)-M06-2X/def2-TZVP level.

**1**
***E* =** -325.542950
***H* =** -325.535433
***G* =** -325.574405
***N*imag =** 0
***SP* =** -325.07774473301

| | | | |
|---|---|---|---|
| C | -1.375848000 | 1.299418000 | -0.000490000 |
| C | -0.009597000 | 1.088777000 | -0.001757000 |
| C | 0.477044000 | -0.220166000 | -0.001204000 |
| C | -1.657513000 | -1.061778000 | 0.001174000 |
| C | -2.226320000 | 0.202199000 | 0.001126000 |
| H | -1.773269000 | 2.306213000 | -0.000907000 |
| H | 0.671714000 | 1.928064000 | -0.003436000 |
| H | -2.287227000 | -1.944935000 | 0.002124000 |
| H | -3.300958000 | 0.319661000 | 0.002110000 |
| N | -0.344501000 | -1.277253000 | -0.000034000 |
| C | 1.916054000 | -0.542592000 | -0.001954000 |
| H | 2.123809000 | -1.607234000 | -0.006053000 |
| C | 2.920192000 | 0.327022000 | 0.002625000 |
| H | 2.767650000 | 1.399072000 | 0.007485000 |
| H | 3.945717000 | -0.017362000 | 0.001792000 |

**2**
***E* =** -386.846091
***H* =** -386.836240
***G* =** -386.880744
***N*imag =** 0
***SP* =** -386.293538965343

| | | | |
|---|---|---|---|
| C | 4.019071000 | 0.563891000 | 0.386712000 |
| H | 3.488913000 | 1.267616000 | 1.018021000 |
| H | 5.090810000 | 0.685568000 | 0.300256000 |
| C | 3.384487000 | -0.420711000 | -0.245098000 |
| H | 3.965896000 | -1.134226000 | -0.821313000 |
| C | 1.937527000 | -0.651495000 | -0.211575000 |
| H | 1.618912000 | -1.682966000 | -0.323662000 |
| C | 1.023407000 | 0.316068000 | -0.090991000 |
| H | 1.364496000 | 1.347666000 | -0.061028000 |
| C | -0.432627000 | 0.139116000 | -0.028958000 |
| C | -1.252080000 | 1.266790000 | -0.122399000 |
| C | -1.042518000 | -1.111153000 | 0.122287000 |
| C | -2.634648000 | 1.153923000 | -0.081762000 |
| H | -0.794394000 | 2.243150000 | -0.232238000 |
| C | -2.422025000 | -1.225323000 | 0.161509000 |
| H | -0.435910000 | -2.002467000 | 0.219672000 |
| C | -3.225668000 | -0.094048000 | 0.057701000 |
| H | -3.249473000 | 2.041812000 | -0.156804000 |
| H | -2.875377000 | -2.201291000 | 0.280684000 |
| H | -4.303424000 | -0.187214000 | 0.091862000 |

**3**
***E* =** -325.541730
***H* =** -325.534205
***G* =** -325.573251
***N*imag =** 0

| | | | |
|---|---|---|---|
| C | -1.785353000 | -0.963895000 | 0.000379000 |

| | | | |
|---|---|---|---|
| N | -2.264732000 | 0.277219000 | 0.000323000 |
| C | -1.371605000 | 1.269147000 | -0.000146000 |
| C | -0.001895000 | 1.074210000 | -0.000517000 |
| C | 0.501097000 | -0.227316000 | -0.000380000 |
| C | -0.430557000 | -1.262377000 | 0.000012000 |
| H | -1.773291000 | 2.276583000 | -0.000248000 |
| H | 0.654291000 | 1.933684000 | -0.000986000 |
| C | 1.938697000 | -0.544489000 | -0.000597000 |
| H | 2.173806000 | -1.604143000 | -0.001804000 |
| C | 2.931945000 | 0.337734000 | 0.000780000 |
| H | 3.962188000 | 0.007768000 | 0.000573000 |
| H | 2.762896000 | 1.407289000 | 0.002202000 |
| H | -0.103997000 | -2.294748000 | 0.000071000 |
| H | -2.516744000 | -1.765052000 | 0.000744000 |

**$BF_3$**
***E* =** -324.597546
***H* =** -324.593091
***G* =** -324.623041
**$N_{imag}$ =** 0
***SP* =** -324.203650773350

| | | | |
|---|---|---|---|
| B | 0.000000000 | 0.000000000 | -0.000192000 |
| F | 0.000000000 | 1.135036000 | -0.655141000 |
| F | 0.000000000 | -1.135036000 | -0.655141000 |
| F | 0.000000000 | 0.000000000 | 1.310389000 |

**1-$BF_3$**
***E* =** -650.185260
***H* =** -650.174043
***G* =** -650.221343
**$N_{imag}$ =** 0
***SP* =** -649.327749744375

| | | | |
|---|---|---|---|
| C | -2.633772000 | -1.046611000 | 0.029853000 |
| C | -2.190430000 | 0.258081000 | 0.125890000 |
| C | -0.828559000 | 0.539644000 | 0.105686000 |
| C | -0.368274000 | -1.745609000 | -0.071945000 |
| C | -1.705475000 | -2.071665000 | -0.071250000 |
| H | -3.693697000 | -1.262650000 | 0.045736000 |
| H | -2.892028000 | 1.070760000 | 0.240043000 |
| H | 0.403529000 | -2.495849000 | -0.145733000 |
| H | -2.003415000 | -3.107074000 | -0.140554000 |
| N | 0.059025000 | -0.477040000 | 0.014238000 |
| C | -0.330760000 | 1.922028000 | 0.200088000 |
| H | 0.649639000 | 2.060511000 | 0.633687000 |
| C | -1.024249000 | 2.969694000 | -0.229047000 |
| H | -1.991343000 | 2.871001000 | -0.706376000 |
| H | -0.623726000 | 3.969318000 | -0.129411000 |
| B | 1.665108000 | -0.203438000 | -0.009786000 |
| F | 2.283622000 | -1.417879000 | -0.215927000 |
| F | 1.917375000 | 0.674319000 | -1.043804000 |
| F | 2.010274000 | 0.332125000 | 1.217090000 |

**3-$BF_3$**
***E* =** -650.188929
***H* =** -650.177394
***G* =** -650.226928
**$N_{imag}$ =** 0

| | | | |
|---|---|---|---|
| C | 0.046402000 | -1.093520000 | 0.029731000 |
| C | 1.419081000 | -1.014992000 | 0.021481000 |
| C | 2.042197000 | 0.234755000 | 0.009339000 |
| C | -0.148584000 | 1.215093000 | 0.017124000 |

| | | | |
|---|---|---|---|
| N | -0.720913000 | 0.005100000 | 0.028591000 |
| H | -0.478200000 | -2.037580000 | 0.041060000 |
| H | 1.988250000 | -1.932520000 | 0.026882000 |
| C | 1.218695000 | 1.362786000 | 0.007958000 |
| C | 3.499839000 | 0.411222000 | -0.001379000 |
| H | 3.840642000 | 1.440560000 | 0.012380000 |
| C | 4.392778000 | -0.572134000 | -0.030334000 |
| H | 4.113906000 | -1.618160000 | -0.048330000 |
| H | 5.451618000 | -0.351466000 | -0.038134000 |
| H | 1.643820000 | 2.356652000 | 0.000273000 |
| H | -0.821430000 | 2.061393000 | 0.019483000 |
| B | -2.331184000 | -0.087195000 | -0.007231000 |
| F | -2.674726000 | -1.390345000 | 0.282547000 |
| F | -2.802816000 | 0.800549000 | 0.940109000 |
| F | -2.728984000 | 0.281147000 | -1.278336000 |

**3-H**
***E* =** -325.971689
***H* =** -325.964049
***G* =** -326.003173
**$N_{imag}$ =** 0

| | | | |
|---|---|---|---|
| C | -1.303206000 | 1.293228000 | -0.008005000 |
| C | 0.049050000 | 1.077178000 | -0.024454000 |
| C | 0.546312000 | -0.232141000 | -0.017306000 |
| C | -1.722951000 | -1.020195000 | 0.016730000 |
| N | -2.147046000 | 0.249913000 | 0.013045000 |
| H | -1.750325000 | 2.275546000 | -0.012458000 |
| H | 0.704377000 | 1.934308000 | -0.046220000 |
| C | -0.376342000 | -1.283588000 | 0.000045000 |
| C | 1.977553000 | -0.544847000 | -0.027799000 |
| H | 2.220309000 | -1.599680000 | -0.082712000 |
| C | 2.955903000 | 0.352571000 | 0.036759000 |
| H | 2.775894000 | 1.417727000 | 0.104051000 |
| H | 3.989899000 | 0.035254000 | 0.029027000 |
| H | -0.041445000 | -2.310907000 | 0.002612000 |
| H | -2.483209000 | -1.786433000 | 0.033810000 |
| H | -3.144093000 | 0.431557000 | 0.024758000 |

**3-COMe**
***E* =** -478.572485
***H* =** -478.561578
***G* =** -478.608933
**$N_{imag}$ =** 0

| | | | |
|---|---|---|---|
| C | 0.283757000 | -1.057758000 | 0.000145000 |
| C | -1.081644000 | -1.010310000 | 0.000094000 |
| C | -1.742998000 | 0.224840000 | -0.000028000 |
| C | 0.413419000 | 1.284571000 | 0.000000000 |
| N | 1.017883000 | 0.077827000 | 0.000072000 |
| H | 0.818905000 | -1.993146000 | 0.000261000 |
| H | -1.620674000 | -1.945075000 | 0.000146000 |
| C | -0.950375000 | 1.379886000 | -0.000050000 |
| C | -3.197616000 | 0.357641000 | -0.000123000 |
| H | -3.572777000 | 1.374090000 | -0.000329000 |
| C | -4.052906000 | -0.661479000 | 0.000029000 |
| H | -3.736743000 | -1.696719000 | 0.000228000 |
| H | -5.119080000 | -0.480058000 | -0.000077000 |
| H | -1.404450000 | 2.360451000 | -0.000122000 |
| H | 1.072698000 | 2.138860000 | -0.000039000 |
| C | 2.506994000 | 0.055120000 | 0.000014000 |
| O | 3.062560000 | 1.102848000 | 0.000149000 |
| C | 3.151902000 | -1.287423000 | -0.000232000 |

| | | | |
|---|---|---|---|
| H | 2.864084000 | -1.852979000 | 0.886357000 |
| H | 2.863884000 | -1.852749000 | -0.886901000 |
| H | 4.225286000 | -1.130779000 | -0.000309000 |

**TS-endo**
***E* =** -712.365530
***H* =** -712.349739
***G* =** -712.408071
**$N_{imag}$ =** 1, -516 $cm^{-1}$
***SP* =** -711.350462344636

| | | | |
|---|---|---|---|
| C | 0.663749000 | 2.633486000 | 0.734382000 |
| C | 0.069660000 | 3.310037000 | -0.319764000 |
| C | -1.109341000 | 2.791049000 | -0.841481000 |
| C | -1.639441000 | 1.639840000 | -0.291974000 |
| C | -0.963771000 | 1.011915000 | 0.765644000 |
| N | 0.177299000 | 1.514853000 | 1.264889000 |
| H | -1.610784000 | 3.283670000 | -1.665215000 |
| H | 1.584217000 | 3.007831000 | 1.171166000 |
| H | 0.513989000 | 4.213689000 | -0.713034000 |
| H | -2.557876000 | 1.218966000 | -0.678740000 |
| C | -1.448044000 | -0.226476000 | 1.362456000 |
| H | -0.787022000 | -0.668898000 | 2.096948000 |
| C | -2.774263000 | -0.628531000 | 1.297769000 |
| H | -3.511893000 | 0.065991000 | 0.917761000 |
| H | -3.138485000 | -1.285998000 | 2.076159000 |
| C | -0.270920000 | -1.636288000 | -0.298179000 |
| H | -0.571224000 | -2.375076000 | 0.433768000 |
| C | -1.109572000 | -1.351581000 | -1.350367000 |
| H | -0.719519000 | -0.805203000 | -2.201586000 |
| C | -2.491044000 | -1.557092000 | -1.285328000 |
| H | -3.108834000 | -1.135117000 | -2.070070000 |
| C | -3.100225000 | -2.050824000 | -0.143755000 |
| H | -2.582118000 | -2.783032000 | 0.460855000 |
| H | -4.179493000 | -2.135171000 | -0.118756000 |
| C | 1.136278000 | -1.243013000 | -0.247193000 |
| C | 2.018779000 | -1.925465000 | 0.595793000 |
| C | 1.629626000 | -0.163463000 | -0.987865000 |
| C | 3.355205000 | -1.564099000 | 0.674730000 |
| H | 1.647127000 | -2.752393000 | 1.190410000 |
| C | 2.964760000 | 0.197875000 | -0.910542000 |
| H | 0.956173000 | 0.421292000 | -1.603710000 |
| C | 3.835038000 | -0.502013000 | -0.082565000 |
| H | 4.022847000 | -2.110847000 | 1.328547000 |
| H | 3.325802000 | 1.039924000 | -1.487915000 |
| H | 4.877154000 | -0.215734000 | -0.021381000 |

**TS-exo**
***E* =** -712.365837
***H* =** -712.349989
***G* =** -712.408544
**$N_{imag}$ =** 1, -512 $cm^{-1}$
***SP* =** -711.349403838126

| | | | |
|---|---|---|---|
| C | -1.863601000 | -2.652685000 | -0.796972000 |
| C | -0.833780000 | -3.496491000 | -0.404790000 |
| C | 0.049134000 | -3.037250000 | 0.563328000 |
| C | -0.131837000 | -1.772019000 | 1.089088000 |
| C | -1.206798000 | -0.991911000 | 0.646256000 |
| N | -2.056932000 | -1.437152000 | -0.293819000 |
| H | 0.873090000 | -3.655678000 | 0.896796000 |
| H | -2.569137000 | -2.973545000 | -1.556833000 |
| H | -0.729776000 | -4.476891000 | -0.848295000 |

| | | | |
|---|---|---|---|
| H | 0.545645000 | -1.374561000 | 1.834227000 |
| C | -1.418523000 | 0.351494000 | 1.174756000 |
| H | -0.661603000 | 0.725586000 | 1.851426000 |
| C | -2.646881000 | 0.986379000 | 1.090630000 |
| H | -2.853685000 | 1.800840000 | 1.769805000 |
| H | -3.502686000 | 0.390045000 | 0.799489000 |
| C | -0.023582000 | 1.375190000 | -0.600377000 |
| H | -0.651336000 | 0.682075000 | -1.147264000 |
| C | -0.447056000 | 2.675121000 | -0.458129000 |
| H | 0.262874000 | 3.436160000 | -0.152708000 |
| C | -1.801309000 | 3.022122000 | -0.520213000 |
| H | -2.081647000 | 4.035006000 | -0.254114000 |
| C | -2.796471000 | 2.067900000 | -0.644757000 |
| H | -2.621577000 | 1.158228000 | -1.206416000 |
| H | -3.830664000 | 2.389123000 | -0.620679000 |
| C | 1.344441000 | 0.913081000 | -0.370628000 |
| C | 1.784103000 | -0.261609000 | -0.988071000 |
| C | 2.230303000 | 1.587247000 | 0.478413000 |
| C | 3.075043000 | -0.731858000 | -0.792928000 |
| H | 1.100661000 | -0.807852000 | -1.629005000 |
| C | 3.518022000 | 1.116783000 | 0.676021000 |
| H | 1.901650000 | 2.477993000 | 0.999738000 |
| C | 3.948338000 | -0.042653000 | 0.037360000 |
| H | 3.396491000 | -1.640924000 | -1.285525000 |
| H | 4.188651000 | 1.651082000 | 1.337141000 |
| H | 4.954297000 | -0.409663000 | 0.195030000 |

**TS′-endo**
***E* =** -712.360728
***H* =** -712.345873
***G* =** -712.403944
**$N_{imag}$ =** 1, -518 $cm^{-1}$

| | | | |
|---|---|---|---|
| C | -4.586027000 | 0.391698000 | -0.496509000 |
| C | -4.359308000 | 1.592209000 | 0.161286000 |
| C | -3.044338000 | 1.954364000 | 0.414810000 |
| C | -2.024298000 | 1.116121000 | 0.001286000 |
| C | -2.345611000 | -0.078019000 | -0.652196000 |
| N | -3.617279000 | -0.429044000 | -0.890446000 |
| H | -2.816366000 | 2.880016000 | 0.928080000 |
| H | -5.600238000 | 0.073632000 | -0.715416000 |
| H | -5.188745000 | 2.217243000 | 0.461209000 |
| H | -0.991025000 | 1.375489000 | 0.188665000 |
| C | -1.309088000 | -1.024853000 | -1.086745000 |
| H | -1.685959000 | -1.895863000 | -1.606892000 |
| C | 0.002329000 | -0.623393000 | -1.288686000 |
| H | 0.260077000 | 0.426195000 | -1.247602000 |
| H | 0.637122000 | -1.223253000 | -1.927624000 |
| C | -1.263368000 | -2.318829000 | 0.743979000 |
| H | -2.272073000 | -2.707819000 | 0.808324000 |
| H | -0.571737000 | -2.926221000 | 0.177812000 |
| C | -0.819763000 | -1.402277000 | 1.669526000 |
| H | -1.517322000 | -1.022603000 | 2.407101000 |
| C | 0.400107000 | -0.740925000 | 1.515876000 |
| H | 0.605659000 | 0.115898000 | 2.148268000 |
| C | 1.245376000 | -1.015410000 | 0.450502000 |
| C | 2.474252000 | -0.245364000 | 0.186636000 |
| C | 2.576997000 | 1.110444000 | 0.509178000 |
| C | 3.562329000 | -0.865580000 | -0.429823000 |
| C | 3.743342000 | 1.813848000 | 0.250885000 |
| H | 1.733235000 | 1.621902000 | 0.957562000 |
| C | 4.730127000 | -0.162570000 | -0.690566000 |

| | | | |
|---|---|---|---|
| H | 3.491184000 | -1.913285000 | -0.699283000 |
| C | 4.826799000 | 1.179485000 | -0.346887000 |
| H | 3.805296000 | 2.863314000 | 0.509691000 |
| H | 5.565206000 | -0.663420000 | -1.163689000 |
| H | 5.735715000 | 1.730311000 | -0.551282000 |
| H | 1.271764000 | -2.033929000 | 0.082882000 |

**TS′-exo**
***E* =** -712.359024
***H* =** -712.344075
***G* =** -712.402410
***N*$_{imag}$ =** 1, -525 cm$^{-1}$

| | | | |
|---|---|---|---|
| C | -4.700769000 | -0.413930000 | 0.413676000 |
| C | -4.547241000 | -1.613304000 | -0.263988000 |
| C | -3.256251000 | -2.028920000 | -0.561110000 |
| C | -2.190132000 | -1.242436000 | -0.166192000 |
| C | -2.437297000 | -0.049505000 | 0.522455000 |
| N | -3.684300000 | 0.355962000 | 0.795144000 |
| H | -3.083043000 | -2.955265000 | -1.094012000 |
| H | -5.692776000 | -0.050672000 | 0.661499000 |
| H | -5.411364000 | -2.196250000 | -0.550347000 |
| H | -1.175495000 | -1.547157000 | -0.389206000 |
| C | -1.349583000 | 0.841323000 | 0.959407000 |
| H | -1.686481000 | 1.726641000 | 1.480137000 |
| C | -0.056575000 | 0.385390000 | 1.162433000 |
| H | 0.160102000 | -0.673279000 | 1.096896000 |
| H | 0.600594000 | 0.942459000 | 1.815087000 |
| C | -1.307374000 | 2.070230000 | -0.948068000 |
| H | -2.259083000 | 2.586769000 | -0.930097000 |
| C | 1.174936000 | 0.740376000 | -0.574006000 |
| C | 2.426873000 | 0.024400000 | -0.268900000 |
| C | 2.628799000 | -1.258791000 | -0.780829000 |
| C | 3.410957000 | 0.574312000 | 0.556110000 |
| C | 3.791445000 | -1.962830000 | -0.501961000 |
| H | 1.865775000 | -1.703314000 | -1.410150000 |
| C | 4.573412000 | -0.128159000 | 0.835684000 |
| H | 3.260787000 | 1.553807000 | 0.994338000 |
| C | 4.770362000 | -1.398236000 | 0.305388000 |
| H | 3.932170000 | -2.953747000 | -0.914725000 |
| H | 5.325367000 | 0.312920000 | 1.477640000 |
| H | 5.676762000 | -1.946126000 | 0.527942000 |
| C | 1.092487000 | 2.121743000 | -0.656061000 |
| C | -0.132137000 | 2.772195000 | -0.822214000 |
| H | 0.458612000 | 0.154142000 | -1.139696000 |
| H | 1.961150000 | 2.715918000 | -0.395140000 |
| H | -1.323370000 | 1.096846000 | -1.417991000 |
| H | -0.171078000 | 3.841422000 | -0.649131000 |

**TS3**
***E* =** -712.365671
***H* =** -712.349876
***G* =** -712.408465
***N*$_{imag}$ =** 1, -509 cm$^{-1}$

| | | | |
|---|---|---|---|
| C | -0.241217000 | 2.813843000 | 0.522580000 |
| N | 0.608531000 | 3.370914000 | -0.343096000 |
| C | 1.704409000 | 2.668505000 | -0.639852000 |
| C | 2.000603000 | 1.428816000 | -0.101257000 |
| C | 1.107018000 | 0.840180000 | 0.804091000 |
| C | -0.046584000 | 1.574559000 | 1.104879000 |
| H | 2.389395000 | 3.124551000 | -1.347209000 |
| H | 2.914808000 | 0.928870000 | -0.391336000 |

| | | | |
|---|---|---|---|
| C | 1.333895000 | -0.469087000 | 1.384650000 |
| H | 0.560244000 | -0.848117000 | 2.041168000 |
| C | 2.574656000 | -1.091450000 | 1.360922000 |
| H | 2.760043000 | -1.868028000 | 2.091365000 |
| H | 3.446222000 | -0.504700000 | 1.102981000 |
| C | 0.019555000 | -1.629790000 | -0.394296000 |
| C | 0.910201000 | -1.349138000 | -1.403025000 |
| H | 0.608677000 | -0.695059000 | -2.212548000 |
| C | 2.260184000 | -1.706871000 | -1.324316000 |
| H | 2.942888000 | -1.289402000 | -2.055726000 |
| C | 2.778445000 | -2.369771000 | -0.222317000 |
| H | 3.840657000 | -2.576915000 | -0.189715000 |
| H | 2.166991000 | -3.100719000 | 0.289494000 |
| H | 0.238516000 | -2.438596000 | 0.290655000 |
| C | -1.336669000 | -1.098325000 | -0.303903000 |
| C | -2.243210000 | -1.677017000 | 0.592523000 |
| C | -1.756233000 | 0.014966000 | -1.041331000 |
| C | -3.530412000 | -1.181904000 | 0.727659000 |
| H | -1.927440000 | -2.528795000 | 1.184487000 |
| C | -3.046243000 | 0.506103000 | -0.911776000 |
| H | -1.063910000 | 0.515540000 | -1.706832000 |
| C | -3.939448000 | -0.089701000 | -0.029843000 |
| H | -4.216564000 | -1.646654000 | 1.424258000 |
| H | -3.350985000 | 1.368246000 | -1.491637000 |
| H | -4.943790000 | 0.299998000 | 0.074305000 |
| H | -0.787436000 | 1.173033000 | 1.785820000 |
| H | -1.131729000 | 3.386842000 | 0.759819000 |

**TS-BF$_3$-endo**
***E* =** -1037.011970
***H* =** -1036.992223
***G* =** -1037.059690
***N*$_{imag}$ =** 1, -510 cm$^{-1}$
***SP* =** -1035.603216600759

| | | | |
|---|---|---|---|
| C | -0.696838000 | -2.076792000 | 1.263231000 |
| C | 0.013891000 | -2.105378000 | 2.435786000 |
| C | 1.315294000 | -1.604343000 | 2.426163000 |
| C | 1.832475000 | -1.087037000 | 1.263462000 |
| C | 1.055933000 | -1.037738000 | 0.090418000 |
| N | -0.200062000 | -1.564777000 | 0.121476000 |
| H | 1.916863000 | -1.621465000 | 3.325612000 |
| H | -1.696885000 | -2.475739000 | 1.194929000 |
| H | -0.431928000 | -2.524710000 | 3.324840000 |
| H | 2.837522000 | -0.694631000 | 1.243766000 |
| C | 1.563474000 | -0.434931000 | -1.112910000 |
| H | 0.879276000 | -0.289990000 | -1.933218000 |
| C | 2.929333000 | -0.252005000 | -1.322772000 |
| H | 3.249723000 | -0.167447000 | -2.353221000 |
| H | 3.630842000 | -0.780907000 | -0.691468000 |
| C | 0.768350000 | 1.920494000 | -0.238601000 |
| C | 1.757120000 | 1.980986000 | 0.708715000 |
| H | 1.477669000 | 2.013238000 | 1.755987000 |
| C | 3.115233000 | 1.836648000 | 0.399144000 |
| H | 3.817985000 | 1.745196000 | 1.219909000 |
| C | 3.558306000 | 1.584755000 | -0.891548000 |
| H | 4.622475000 | 1.478140000 | -1.060046000 |
| H | 3.017808000 | 2.014645000 | -1.724668000 |
| B | -1.071061000 | -1.746613000 | -1.229626000 |
| F | -0.324514000 | -2.544050000 | -2.083390000 |
| F | -2.254222000 | -2.372130000 | -0.879201000 |
| F | -1.321503000 | -0.511401000 | -1.792881000 |

| | | | |
|---|---|---|---|
| H | 1.011675000 | 2.092424000 | -1.279861000 |
| C | -0.656353000 | 1.881764000 | 0.077412000 |
| C | -1.582899000 | 2.400059000 | -0.832186000 |
| C | -1.129120000 | 1.311809000 | 1.263313000 |
| C | -2.939220000 | 2.376502000 | -0.554283000 |
| H | -1.226660000 | 2.822956000 | -1.764336000 |
| C | -2.487922000 | 1.272876000 | 1.535162000 |
| H | -0.428407000 | 0.871845000 | 1.964467000 |
| C | -3.396838000 | 1.809677000 | 0.630103000 |
| H | -3.643310000 | 2.789706000 | -1.265181000 |
| H | -2.840522000 | 0.817973000 | 2.452288000 |
| H | -4.457549000 | 1.779430000 | 0.843596000 |

**TS-$BF_3$-exo**
***E*** **=** -1037.009563
***H*** **=** -1036.989830
***G*** **=** -1037.056390
$N_{imag}$ **=** 1, -525 $cm^{-1}$
***SP*** **=** -1035.600177693532

| | | | |
|---|---|---|---|
| C | -1.334823000 | -2.167932000 | 0.775669000 |
| C | -0.890627000 | -2.582001000 | 2.004281000 |
| C | 0.458570000 | -2.397970000 | 2.309430000 |
| C | 1.282036000 | -1.794752000 | 1.391562000 |
| C | 0.783173000 | -1.358215000 | 0.150030000 |
| N | -0.530524000 | -1.580469000 | -0.131536000 |
| H | 0.854548000 | -2.720888000 | 3.263408000 |
| H | -2.360428000 | -2.299089000 | 0.467438000 |
| H | -1.577266000 | -3.043415000 | 2.697490000 |
| H | 2.325315000 | -1.635060000 | 1.622447000 |
| C | 1.614038000 | -0.659770000 | -0.792977000 |
| H | 1.125553000 | -0.195745000 | -1.633472000 |
| C | 3.006653000 | -0.734474000 | -0.780859000 |
| H | 3.510232000 | -0.505692000 | -1.709212000 |
| H | 3.481173000 | -1.528712000 | -0.215973000 |
| C | 1.146891000 | 1.608935000 | 0.504967000 |
| H | 1.478153000 | 0.890334000 | 1.243463000 |
| C | 2.098471000 | 2.307720000 | -0.192530000 |
| H | 1.807914000 | 3.153104000 | -0.804582000 |
| C | 3.428334000 | 1.874774000 | -0.265533000 |
| H | 4.092099000 | 2.392718000 | -0.948575000 |
| C | 3.865294000 | 0.686828000 | 0.304475000 |
| H | 3.419937000 | 0.349567000 | 1.232841000 |
| H | 4.909454000 | 0.422567000 | 0.195345000 |
| C | -0.284160000 | 1.884000000 | 0.538107000 |
| C | -1.081186000 | 1.154989000 | 1.425947000 |
| C | -0.902471000 | 2.829170000 | -0.288762000 |
| C | -2.452246000 | 1.354481000 | 1.486178000 |
| H | -0.614048000 | 0.422646000 | 2.076606000 |
| C | -2.268273000 | 3.037150000 | -0.221485000 |
| H | -0.314565000 | 3.398647000 | -0.996772000 |
| C | -3.050363000 | 2.298108000 | 0.662754000 |
| H | -3.051620000 | 0.774688000 | 2.176659000 |
| H | -2.731057000 | 3.772941000 | -0.866650000 |
| H | -4.119709000 | 2.459554000 | 0.706187000 |
| B | -1.143047000 | -1.277506000 | -1.599489000 |
| F | -0.395003000 | -2.007908000 | -2.508512000 |
| F | -2.460343000 | -1.701816000 | -1.592349000 |
| F | -1.069394000 | 0.075989000 | -1.860914000 |

**TS′-$BF_3$-endo**
***E*** **=** -1037.006814

***H* =** -1036.987144
***G* =** -1037.054661
***N*$_{imag}$ =** 1, -510 cm$^{-1}$
***SP* =** -1035.59790747696

| | | | |
|---|---|---|---|
| C | -3.606861000 | 1.409922000 | -0.290914000 |
| C | -3.098628000 | 2.682481000 | -0.204288000 |
| C | -1.717433000 | 2.835322000 | -0.158453000 |
| C | -0.912134000 | 1.719279000 | -0.198897000 |
| C | -1.472265000 | 0.439520000 | -0.273497000 |
| N | -2.822355000 | 0.317513000 | -0.325344000 |
| H | -1.275626000 | 3.820731000 | -0.089361000 |
| H | -4.667675000 | 1.220910000 | -0.340827000 |
| H | -3.769248000 | 3.527866000 | -0.179165000 |
| H | 0.161581000 | 1.821038000 | -0.153293000 |
| C | -0.626339000 | -0.748113000 | -0.266172000 |
| H | -1.119033000 | -1.706030000 | -0.277945000 |
| C | 0.694010000 | -0.673045000 | -0.694404000 |
| H | 1.066825000 | 0.228305000 | -1.160783000 |
| H | 1.162802000 | -1.584562000 | -1.040318000 |
| C | -0.525183000 | -0.957279000 | 2.015899000 |
| H | -1.559028000 | -1.040107000 | 2.324164000 |
| H | -0.042767000 | -1.890699000 | 1.763735000 |
| C | 0.203400000 | 0.166653000 | 2.320701000 |
| H | -0.289517000 | 0.990428000 | 2.823783000 |
| C | 1.469731000 | 0.392481000 | 1.776737000 |
| H | 1.911486000 | 1.375637000 | 1.897246000 |
| C | 2.073528000 | -0.523699000 | 0.925319000 |
| B | -3.542370000 | -1.128462000 | -0.467428000 |
| F | -3.233234000 | -1.875798000 | 0.655838000 |
| F | -3.063762000 | -1.723184000 | -1.620471000 |
| F | -4.902726000 | -0.906833000 | -0.551101000 |
| C | 3.360799000 | -0.265278000 | 0.251531000 |
| C | 3.759358000 | 1.024165000 | -0.109046000 |
| C | 4.194143000 | -1.335690000 | -0.076108000 |
| C | 4.967198000 | 1.236494000 | -0.755327000 |
| H | 3.116372000 | 1.869302000 | 0.106821000 |
| C | 5.403176000 | -1.124754000 | -0.724002000 |
| H | 3.890516000 | -2.342887000 | 0.186099000 |
| C | 5.795686000 | 0.163187000 | -1.063410000 |
| H | 5.260606000 | 2.242567000 | -1.026770000 |
| H | 6.038053000 | -1.967738000 | -0.965121000 |
| H | 6.737224000 | 0.330359000 | -1.570147000 |
| H | 1.870164000 | -1.572931000 | 1.104006000 |

**TS′-BF$_3$-exo**
***E* =** -1037.004667
***H* =** -1036.985720
***G* =** -1037.053105
***N*$_{imag}$ =** 1, -513 cm$^{-1}$

| | | | |
|---|---|---|---|
| C | -3.661509000 | 1.528016000 | 0.083316000 |
| C | -3.181455000 | 2.756699000 | 0.460173000 |
| C | -1.803303000 | 2.921726000 | 0.552461000 |
| C | -0.976441000 | 1.858777000 | 0.269806000 |
| C | -1.506814000 | 0.620032000 | -0.107991000 |
| N | -2.853757000 | 0.487121000 | -0.194257000 |
| H | -1.381552000 | 3.873208000 | 0.848678000 |
| H | -4.718192000 | 1.334615000 | -0.014689000 |
| H | -3.870265000 | 3.559738000 | 0.673921000 |
| H | 0.095539000 | 1.971578000 | 0.347691000 |
| C | -0.640019000 | -0.523943000 | -0.373448000 |
| H | -1.122326000 | -1.463644000 | -0.579244000 |

| | | | |
|---|---|---|---|
| C | 0.672815000 | -0.344591000 | -0.790918000 |
| H | 1.031696000 | 0.643270000 | -1.049325000 |
| H | 1.150814000 | -1.149451000 | -1.330793000 |
| C | -0.520970000 | -1.167706000 | 1.854389000 |
| C | 2.041808000 | -0.449567000 | 0.833919000 |
| B | -3.546441000 | -0.899780000 | -0.674533000 |
| F | -3.231612000 | -1.880468000 | 0.248886000 |
| F | -3.052172000 | -1.201724000 | -1.929523000 |
| F | -4.910045000 | -0.686820000 | -0.717815000 |
| C | 3.334065000 | -0.084223000 | 0.222857000 |
| C | 3.817179000 | 1.218559000 | 0.355146000 |
| C | 4.077390000 | -0.999646000 | -0.525393000 |
| C | 5.023405000 | 1.591246000 | -0.220487000 |
| H | 3.242318000 | 1.942229000 | 0.922232000 |
| C | 5.282457000 | -0.628163000 | -1.101495000 |
| H | 3.703955000 | -2.006357000 | -0.669779000 |
| C | 5.761965000 | 0.667812000 | -0.948777000 |
| H | 5.386905000 | 2.603853000 | -0.101069000 |
| H | 5.846345000 | -1.349768000 | -1.678726000 |
| H | 6.701439000 | 0.957412000 | -1.401476000 |
| C | 1.766931000 | -1.725363000 | 1.304465000 |
| C | 0.502739000 | -2.078723000 | 1.783961000 |
| H | -0.317517000 | -0.118509000 | 2.016224000 |
| H | -1.530155000 | -1.487658000 | 2.078589000 |
| H | 0.283391000 | -3.131542000 | 1.918723000 |
| H | 2.487918000 | -2.515259000 | 1.126005000 |
| H | 1.521187000 | 0.380782000 | 1.299671000 |

**TS3-$BF_3$**
***E* =** -1037.019605
***H* =** -1036.999594
***G* =** -1037.068777
**$N_{imag}$ =** 1, -480 $cm^{-1}$

| | | | |
|---|---|---|---|
| C | 1.510288000 | 0.120056000 | 1.113205000 |
| N | 2.101291000 | -0.762780000 | 0.287098000 |
| C | 1.397420000 | -1.816822000 | -0.157086000 |
| C | 0.091752000 | -2.025063000 | 0.206442000 |
| C | -0.560784000 | -1.108782000 | 1.055899000 |
| C | 0.208107000 | -0.017405000 | 1.508960000 |
| H | 1.925285000 | -2.490487000 | -0.816349000 |
| H | -0.418674000 | -2.893776000 | -0.182115000 |
| C | -1.938029000 | -1.242205000 | 1.425547000 |
| H | -2.360020000 | -0.450863000 | 2.032684000 |
| C | -2.682118000 | -2.392124000 | 1.185608000 |
| H | -3.576288000 | -2.536693000 | 1.778247000 |
| H | -2.162473000 | -3.309512000 | 0.943306000 |
| C | -2.493092000 | 0.321685000 | -0.631872000 |
| C | -2.214930000 | -0.656171000 | -1.549884000 |
| H | -1.401465000 | -0.515697000 | -2.251043000 |
| C | -2.815790000 | -1.922136000 | -1.506694000 |
| H | -2.419935000 | -2.690458000 | -2.161383000 |
| C | -3.728275000 | -2.287979000 | -0.528350000 |
| H | -4.142086000 | -3.287805000 | -0.558764000 |
| H | -4.397000000 | -1.541116000 | -0.121116000 |
| H | -3.401815000 | 0.252974000 | -0.047441000 |
| C | -1.740809000 | 1.554580000 | -0.450461000 |
| C | -2.236176000 | 2.523307000 | 0.431188000 |
| C | -0.503576000 | 1.787035000 | -1.064902000 |
| C | -1.534646000 | 3.692234000 | 0.675914000 |
| H | -3.183975000 | 2.347305000 | 0.927157000 |
| C | 0.195219000 | 2.959244000 | -0.824616000 |

| | | | |
|---|---|---|---|
| H | -0.073276000 | 1.043431000 | -1.724438000 |
| C | -0.316214000 | 3.916156000 | 0.044486000 |
| H | -1.935711000 | 4.428638000 | 1.360291000 |
| H | 1.150981000 | 3.120963000 | -1.306757000 |
| H | 0.235145000 | 4.827738000 | 0.235144000 |
| H | -0.228203000 | 0.726624000 | 2.161909000 |
| H | 2.124571000 | 0.949725000 | 1.436100000 |
| B | 3.597905000 | -0.479393000 | -0.187159000 |
| F | 4.353123000 | -0.222495000 | 0.945526000 |
| F | 4.049109000 | -1.598020000 | -0.861923000 |
| F | 3.568466000 | 0.634045000 | -1.014674000 |

**TS3-H**
***E* =** -712.806574
***H* =** -712.790542
***G* =** -712.848972
**$N_{imag}$ =** 1, -450 $cm^{-1}$

| | | | |
|---|---|---|---|
| C | -1.452020000 | 2.028384000 | 0.740461000 |
| N | -1.179954000 | 2.685536000 | -0.404858000 |
| C | 0.030738000 | 2.606153000 | -0.993977000 |
| C | 1.028113000 | 1.864824000 | -0.433414000 |
| C | 0.798808000 | 1.148211000 | 0.768057000 |
| C | -0.494322000 | 1.265291000 | 1.337113000 |
| H | 0.152566000 | 3.160410000 | -1.912542000 |
| H | 1.983575000 | 1.828136000 | -0.935739000 |
| C | 1.782620000 | 0.338946000 | 1.397811000 |
| H | 1.462932000 | -0.214088000 | 2.273453000 |
| C | 3.127461000 | 0.305801000 | 1.047970000 |
| H | 3.826214000 | -0.018266000 | 1.808994000 |
| H | 3.524686000 | 1.055962000 | 0.377363000 |
| C | 0.664355000 | -1.582756000 | -0.273095000 |
| C | 1.468501000 | -1.222468000 | -1.316637000 |
| H | 1.021565000 | -0.864387000 | -2.235958000 |
| C | 2.867574000 | -1.122208000 | -1.213209000 |
| H | 3.398473000 | -0.693808000 | -2.056343000 |
| C | 3.570032000 | -1.329611000 | -0.039869000 |
| H | 4.648407000 | -1.242476000 | -0.065786000 |
| H | 3.188516000 | -2.010405000 | 0.709554000 |
| H | 1.100856000 | -2.065139000 | 0.593118000 |
| C | -0.781008000 | -1.426903000 | -0.234416000 |
| C | -1.480193000 | -1.888275000 | 0.886642000 |
| C | -1.494103000 | -0.746803000 | -1.230521000 |
| C | -2.847103000 | -1.691946000 | 1.005834000 |
| H | -0.936212000 | -2.398658000 | 1.673308000 |
| C | -2.859621000 | -0.551702000 | -1.111269000 |
| H | -0.980716000 | -0.355930000 | -2.099370000 |
| C | -3.541796000 | -1.023056000 | 0.006080000 |
| H | -3.369737000 | -2.056377000 | 1.880698000 |
| H | -3.395278000 | -0.023431000 | -1.889649000 |
| H | -4.608409000 | -0.864141000 | 0.098011000 |
| H | -0.733649000 | 0.744949000 | 2.253276000 |
| H | -2.449403000 | 2.143664000 | 1.137402000 |
| H | -1.902930000 | 3.243963000 | -0.837164000 |

**TS3-COMe**
***E* =** -865.414308
***H* =** -865.394860
***G* =** -865.461056
**$N_{imag}$ =** 1, -401 $cm^{-1}$

| | | | |
|---|---|---|---|
| C | 1.395125000 | -0.626110000 | 1.273014000 |
| N | 1.685210000 | -1.399052000 | 0.184719000 |

| | | | |
|---|---|---|---|
| C | 0.664239000 | -2.009891000 | -0.491856000 |
| C | -0.626627000 | -1.874906000 | -0.105680000 |
| C | -0.970987000 | -1.065072000 | 1.013908000 |
| C | 0.120102000 | -0.456836000 | 1.695456000 |
| H | 0.967276000 | -2.597636000 | -1.344065000 |
| H | -1.386165000 | -2.379474000 | -0.684849000 |
| C | -2.290454000 | -0.850817000 | 1.461214000 |
| H | -2.413792000 | -0.168764000 | 2.294564000 |
| C | -3.419937000 | -1.470273000 | 0.946510000 |
| H | -4.310411000 | -1.481331000 | 1.561849000 |
| H | -3.325895000 | -2.317435000 | 0.281142000 |
| C | -1.853367000 | 1.308014000 | -0.491778000 |
| C | -2.323930000 | 0.503465000 | -1.483227000 |
| H | -1.689433000 | 0.273829000 | -2.330389000 |
| C | -3.555640000 | -0.186831000 | -1.418230000 |
| H | -3.770050000 | -0.881000000 | -2.223570000 |
| C | -4.406237000 | -0.181575000 | -0.335327000 |
| H | -5.342405000 | -0.718451000 | -0.409584000 |
| H | -4.393328000 | 0.631466000 | 0.378106000 |
| H | -2.521421000 | 1.618058000 | 0.304008000 |
| C | -0.495262000 | 1.816135000 | -0.392989000 |
| C | -0.170206000 | 2.657200000 | 0.678072000 |
| C | 0.525585000 | 1.426061000 | -1.270161000 |
| C | 1.128916000 | 3.100356000 | 0.868561000 |
| H | -0.950122000 | 2.953219000 | 1.370145000 |
| C | 1.823809000 | 1.867176000 | -1.077649000 |
| H | 0.312047000 | 0.767584000 | -2.101999000 |
| C | 2.130805000 | 2.704152000 | -0.007803000 |
| H | 1.361462000 | 3.749157000 | 1.702661000 |
| H | 2.602424000 | 1.558098000 | -1.764281000 |
| H | 3.147619000 | 3.045506000 | 0.138942000 |
| H | -0.061926000 | 0.168228000 | 2.558265000 |
| H | 2.223874000 | -0.153412000 | 1.774796000 |
| C | 3.031858000 | -1.554882000 | -0.320135000 |
| O | 3.201453000 | -2.231474000 | -1.291406000 |
| C | 4.120080000 | -0.841447000 | 0.417202000 |
| H | 4.180935000 | -1.187359000 | 1.449152000 |
| H | 5.053631000 | -1.052917000 | -0.093272000 |
| H | 3.940349000 | 0.234276000 | 0.421993000 |

**2-endo**
***E* =** -712.435856
***H* =** -712.420659
***G* =** -712.478093
**$N_{imag}$ =** 0
***SP* =** -711.429259103841

| | | | |
|---|---|---|---|
| C | -1.162069000 | 2.644299000 | -0.922872000 |
| C | -0.923196000 | 3.342300000 | 0.249660000 |
| C | 0.118271000 | 2.913895000 | 1.060002000 |
| C | 0.863592000 | 1.815685000 | 0.668562000 |
| C | 0.541517000 | 1.160273000 | -0.520816000 |
| N | -0.454973000 | 1.580591000 | -1.303931000 |
| H | 0.345967000 | 3.429204000 | 1.984564000 |
| H | -1.959753000 | 2.952856000 | -1.590401000 |
| H | -1.530787000 | 4.196959000 | 0.513251000 |
| H | 1.678861000 | 1.454834000 | 1.281677000 |
| C | 1.256821000 | -0.096624000 | -0.959488000 |
| H | 0.921420000 | -0.283466000 | -1.979826000 |
| C | 2.796169000 | 0.045832000 | -0.936745000 |
| H | 3.070867000 | 0.956423000 | -0.403278000 |
| H | 3.164675000 | 0.161472000 | -1.956420000 |

| | | | |
|---|---|---|---|
| C | 0.777895000 | -1.340417000 | -0.120098000 |
| H | 1.045404000 | -2.201905000 | -0.742877000 |
| C | 1.565114000 | -1.462195000 | 1.156798000 |
| H | 1.051048000 | -1.643392000 | 2.093461000 |
| C | 2.890395000 | -1.390806000 | 1.085734000 |
| H | 3.512007000 | -1.501645000 | 1.966408000 |
| C | 3.516209000 | -1.127613000 | -0.252440000 |
| H | 3.429593000 | -2.021470000 | -0.881175000 |
| H | 4.579216000 | -0.908548000 | -0.160913000 |
| C | -0.721913000 | -1.360479000 | 0.044218000 |
| C | -1.516409000 | -1.948984000 | -0.936637000 |
| C | -1.350969000 | -0.749248000 | 1.127540000 |
| C | -2.901864000 | -1.924128000 | -0.846465000 |
| H | -1.041187000 | -2.429906000 | -1.784777000 |
| C | -2.736151000 | -0.721002000 | 1.222781000 |
| H | -0.755130000 | -0.277622000 | 1.900031000 |
| C | -3.517377000 | -1.306712000 | 0.234922000 |
| H | -3.500322000 | -2.388693000 | -1.620049000 |
| H | -3.206215000 | -0.238730000 | 2.070727000 |
| H | -4.597050000 | -1.284960000 | 0.309182000 |

**2-exo**
***E* =** -712.439492
***H* =** -712.424058
***G* =** -712.483056
**$N_{imag}$ =** 0
***SP* =** -711.431863387295

| | | | |
|---|---|---|---|
| C | 1.135396000 | 2.982485000 | -0.918181000 |
| C | 0.238126000 | 3.742800000 | -0.181994000 |
| C | -0.378175000 | 3.148907000 | 0.908529000 |
| C | -0.075634000 | 1.830982000 | 1.210956000 |
| C | 0.836356000 | 1.143075000 | 0.415388000 |
| N | 1.431182000 | 1.716595000 | -0.633513000 |
| H | -1.085285000 | 3.703034000 | 1.512736000 |
| H | 1.638847000 | 3.412560000 | -1.777736000 |
| H | 0.034324000 | 4.768151000 | -0.458083000 |
| H | -0.543944000 | 1.329565000 | 2.048393000 |
| C | 1.155285000 | -0.310600000 | 0.665327000 |
| H | 0.698236000 | -0.592502000 | 1.615811000 |
| C | 2.676453000 | -0.581107000 | 0.741939000 |
| H | 2.917063000 | -0.921649000 | 1.749683000 |
| H | 3.222283000 | 0.347087000 | 0.575649000 |
| C | 0.448063000 | -1.176575000 | -0.427993000 |
| H | 0.762911000 | -0.755843000 | -1.390415000 |
| C | 0.940441000 | -2.597920000 | -0.349335000 |
| H | 0.220508000 | -3.408235000 | -0.374937000 |
| C | 2.247851000 | -2.820363000 | -0.279784000 |
| H | 2.647711000 | -3.826876000 | -0.243007000 |
| C | 3.172992000 | -1.639967000 | -0.255906000 |
| H | 3.208359000 | -1.192236000 | -1.255635000 |
| H | 4.191837000 | -1.935024000 | -0.007335000 |
| C | -1.049713000 | -1.039339000 | -0.316972000 |
| C | -1.754286000 | -0.188578000 | -1.164329000 |
| C | -1.750527000 | -1.715406000 | 0.680755000 |
| C | -3.125803000 | -0.017241000 | -1.022874000 |
| H | -1.219696000 | 0.349125000 | -1.939968000 |
| C | -3.120994000 | -1.549251000 | 0.825522000 |
| H | -1.215548000 | -2.375701000 | 1.355155000 |
| C | -3.813712000 | -0.698013000 | -0.026954000 |
| H | -3.657444000 | 0.648348000 | -1.691314000 |
| H | -3.648776000 | -2.082927000 | 1.605956000 |

| | | | |
|---|---|---|---|
| H | -4.882366000 | -0.566377000 | 0.085078000 |

**2′-endo**
***E* =** -712.436309
***H* =** -712.421939
***G* =** -712.480437
**$N_{imag}$ =** 0

| | | | |
|---|---|---|---|
| C | -4.561132000 | 0.518121000 | -0.899920000 |
| C | -4.543398000 | 1.555791000 | 0.017568000 |
| C | -3.376116000 | 1.764783000 | 0.737855000 |
| C | -2.288676000 | 0.940341000 | 0.506628000 |
| C | -2.395073000 | -0.082557000 | -0.437434000 |
| N | -3.521858000 | -0.285238000 | -1.124684000 |
| H | -3.312640000 | 2.561209000 | 1.468652000 |
| H | -5.453178000 | 0.319434000 | -1.484665000 |
| H | -5.416522000 | 2.177497000 | 0.159446000 |
| H | -1.366040000 | 1.079376000 | 1.054116000 |
| C | -1.272779000 | -1.065616000 | -0.687218000 |
| H | -1.527861000 | -1.573513000 | -1.616483000 |
| C | 0.110853000 | -0.394229000 | -0.835655000 |
| H | 0.045816000 | 0.664766000 | -0.580567000 |
| H | 0.437001000 | -0.440136000 | -1.875843000 |
| C | -1.282503000 | -2.141671000 | 0.434064000 |
| H | -2.310841000 | -2.407493000 | 0.678267000 |
| H | -0.809857000 | -3.044200000 | 0.031883000 |
| C | -0.517579000 | -1.688417000 | 1.642459000 |
| H | -0.941759000 | -1.812127000 | 2.631782000 |
| C | 0.685316000 | -1.153644000 | 1.463932000 |
| H | 1.294354000 | -0.819928000 | 2.296643000 |
| C | 1.211156000 | -1.017323000 | 0.058397000 |
| C | 2.484941000 | -0.215655000 | -0.040148000 |
| C | 2.595783000 | 1.019267000 | 0.598471000 |
| C | 3.560224000 | -0.671518000 | -0.796887000 |
| C | 3.753387000 | 1.776189000 | 0.487450000 |
| H | 1.765096000 | 1.390695000 | 1.189807000 |
| C | 4.721697000 | 0.082990000 | -0.912049000 |
| H | 3.487626000 | -1.630110000 | -1.298393000 |
| C | 4.821995000 | 1.309029000 | -0.268991000 |
| H | 3.823364000 | 2.731622000 | 0.992194000 |
| H | 5.549565000 | -0.289479000 | -1.502210000 |
| H | 5.727006000 | 1.896871000 | -0.353651000 |
| H | 1.413291000 | -2.023107000 | -0.329313000 |

**2′-exo**
***E* =** -712.436762
***H* =** -712.422391
***G* =** -712.480353
**$N_{imag}$ =** 0

| | | | |
|---|---|---|---|
| C | -4.650191000 | -0.461075000 | 0.806758000 |
| C | -4.658898000 | -1.580275000 | -0.009136000 |
| C | -3.494767000 | -1.893666000 | -0.697377000 |
| C | -2.385160000 | -1.083858000 | -0.535392000 |
| C | -2.464251000 | 0.022343000 | 0.312147000 |
| N | -3.586156000 | 0.325917000 | 0.967500000 |
| H | -3.453991000 | -2.757905000 | -1.348186000 |
| H | -5.539402000 | -0.181789000 | 1.361793000 |
| H | -5.550336000 | -2.185481000 | -0.098489000 |
| H | -1.462715000 | -1.302549000 | -1.059031000 |
| C | -1.301085000 | 0.969268000 | 0.484855000 |
| H | -1.510964000 | 1.543929000 | 1.386535000 |
| C | 0.063938000 | 0.263621000 | 0.656161000 |

| | | | |
|---|---|---|---|
| H | -0.062208000 | -0.818359000 | 0.725033000 |
| H | 0.514551000 | 0.585530000 | 1.596525000 |
| C | -1.295467000 | 1.973481000 | -0.697105000 |
| H | -2.161938000 | 2.629468000 | -0.619002000 |
| C | 1.075881000 | 0.582485000 | -0.469365000 |
| C | 2.407460000 | -0.062210000 | -0.179484000 |
| C | 2.783323000 | -1.231555000 | -0.834283000 |
| C | 3.268583000 | 0.470742000 | 0.779123000 |
| C | 3.990477000 | -1.855491000 | -0.545095000 |
| H | 2.122251000 | -1.656498000 | -1.581373000 |
| C | 4.476302000 | -0.148404000 | 1.071231000 |
| H | 2.990048000 | 1.379156000 | 1.302516000 |
| C | 4.841394000 | -1.314682000 | 0.409155000 |
| H | 4.266708000 | -2.762330000 | -1.068084000 |
| H | 5.134854000 | 0.280016000 | 1.816359000 |
| H | 5.783822000 | -1.796778000 | 0.635009000 |
| C | 1.134287000 | 2.076462000 | -0.657186000 |
| C | -0.009630000 | 2.743796000 | -0.758731000 |
| H | 0.685654000 | 0.141946000 | -1.395564000 |
| H | 2.097040000 | 2.572085000 | -0.713001000 |
| H | -1.413209000 | 1.404947000 | -1.628057000 |
| H | -0.030243000 | 3.817309000 | -0.903146000 |

**4-endo**
***E* =** -712.434881
***H* =** -712.419665
***G* =** -712.477602
**$N_{imag}$ =** 0

| | | | |
|---|---|---|---|
| C | 0.722718000 | 2.927786000 | -0.769475000 |
| N | 0.183392000 | 3.553764000 | 0.275917000 |
| C | -0.815007000 | 2.928960000 | 0.899880000 |
| C | -1.305730000 | 1.688677000 | 0.521162000 |
| C | -0.734338000 | 1.029157000 | -0.564153000 |
| C | 0.304968000 | 1.683725000 | -1.217229000 |
| H | -1.250337000 | 3.445584000 | 1.748861000 |
| H | -2.119795000 | 1.244404000 | 1.078381000 |
| C | -1.160644000 | -0.354743000 | -0.991511000 |
| H | -0.730369000 | -0.526238000 | -1.979803000 |
| C | -2.698598000 | -0.517914000 | -1.068661000 |
| H | -2.993060000 | -0.677472000 | -2.106115000 |
| H | -3.176415000 | 0.409300000 | -0.752059000 |
| C | -0.520589000 | -1.446444000 | -0.055951000 |
| C | -1.295746000 | -1.544240000 | 1.229367000 |
| H | -0.773583000 | -1.541742000 | 2.178667000 |
| C | -2.617019000 | -1.664302000 | 1.154740000 |
| H | -3.228024000 | -1.756976000 | 2.044861000 |
| C | -3.255894000 | -1.662805000 | -0.202186000 |
| H | -4.339679000 | -1.577695000 | -0.133732000 |
| H | -3.046798000 | -2.617818000 | -0.697447000 |
| H | -0.669406000 | -2.386783000 | -0.599999000 |
| C | 0.967658000 | -1.250960000 | 0.093921000 |
| C | 1.829083000 | -1.800704000 | -0.853320000 |
| C | 1.513747000 | -0.479591000 | 1.117930000 |
| C | 3.199276000 | -1.587175000 | -0.784533000 |
| H | 1.417968000 | -2.404054000 | -1.655625000 |
| C | 2.883821000 | -0.264893000 | 1.192993000 |
| H | 0.862580000 | -0.025972000 | 1.856233000 |
| C | 3.731656000 | -0.816639000 | 0.241116000 |
| H | 3.851554000 | -2.025172000 | -1.529523000 |
| H | 3.289270000 | 0.339301000 | 1.994825000 |
| H | 4.799400000 | -0.648934000 | 0.299290000 |

| | | | |
|---|---|---|---|
| H | 0.795186000 | 1.223201000 | -2.066496000 |
| H | 1.531591000 | 3.443359000 | -1.276284000 |

**2-$BF_3$-endo**

***E*** **=** -1037.078701
***H*** **=** -1037.059541
***G*** **=** -1037.125935
$N_{imag}$ **=** 0
***SP*** **=** -1035.678491769

| | | | |
|---|---|---|---|
| C | -2.401395000 | 0.379794000 | 1.433141000 |
| C | -1.920346000 | 0.455490000 | 2.717710000 |
| C | -0.671219000 | -0.086354000 | 2.986306000 |
| C | 0.048832000 | -0.651214000 | 1.954319000 |
| C | -0.462098000 | -0.672979000 | 0.660607000 |
| N | -1.694956000 | -0.177205000 | 0.431084000 |
| H | -0.264206000 | -0.064126000 | 3.988548000 |
| H | -3.377146000 | 0.753057000 | 1.165334000 |
| H | -2.522104000 | 0.914827000 | 3.487591000 |
| H | 1.026870000 | -1.079153000 | 2.128570000 |
| C | 0.365727000 | -1.238635000 | -0.465556000 |
| H | -0.160406000 | -1.075905000 | -1.399274000 |
| C | 0.573156000 | -2.759546000 | -0.267342000 |
| H | -0.167774000 | -3.296870000 | -0.859050000 |
| H | 0.395401000 | -3.021023000 | 0.777124000 |
| C | 1.735682000 | -0.471596000 | -0.627240000 |
| C | 2.831602000 | -1.087736000 | 0.200296000 |
| H | 3.508781000 | -0.451680000 | 0.757982000 |
| C | 2.981693000 | -2.407575000 | 0.165475000 |
| H | 3.781662000 | -2.899615000 | 0.705648000 |
| C | 1.994439000 | -3.219442000 | -0.620199000 |
| H | 2.099558000 | -4.283516000 | -0.414129000 |
| H | 2.167069000 | -3.082874000 | -1.694039000 |
| B | -2.429041000 | -0.319403000 | -1.018303000 |
| F | -2.403688000 | -1.662855000 | -1.346114000 |
| F | -3.724505000 | 0.129754000 | -0.864408000 |
| F | -1.744479000 | 0.447675000 | -1.937908000 |
| H | 1.998297000 | -0.665583000 | -1.673271000 |
| C | 1.574645000 | 1.025329000 | -0.496903000 |
| C | 1.109909000 | 1.755485000 | -1.589171000 |
| C | 1.837384000 | 1.705988000 | 0.690137000 |
| C | 0.907207000 | 3.125186000 | -1.500736000 |
| H | 0.897841000 | 1.239336000 | -2.518450000 |
| C | 1.636813000 | 3.077804000 | 0.784087000 |
| H | 2.200961000 | 1.166601000 | 1.556174000 |
| C | 1.169478000 | 3.791971000 | -0.310498000 |
| H | 0.547684000 | 3.672576000 | -2.362911000 |
| H | 1.848108000 | 3.587727000 | 1.715484000 |
| H | 1.014296000 | 4.860692000 | -0.238129000 |

**2-$BF_3$-exo**

***E*** **=** -1037.089608
***H*** **=** -1037.070422
***G*** **=** -1037.137077
$N_{imag}$ **=** 0
***SP*** **=** -1035.689913718135

| | | | |
|---|---|---|---|
| C | 2.641994000 | 1.540834000 | -0.385533000 |
| C | 2.662479000 | 1.979475000 | -1.688420000 |
| C | 1.815491000 | 1.369304000 | -2.600163000 |
| C | 0.971353000 | 0.365843000 | -2.165077000 |
| C | 0.966596000 | -0.029138000 | -0.832806000 |
| N | 1.822433000 | 0.559715000 | 0.030817000 |

| | | | |
|---|---|---|---|
| H | 1.811768000 | 1.671970000 | -3.638930000 |
| H | 3.290133000 | 1.960737000 | 0.367507000 |
| H | 3.335376000 | 2.773501000 | -1.975290000 |
| H | 0.308207000 | -0.124604000 | -2.862583000 |
| C | 0.059615000 | -1.137234000 | -0.364379000 |
| H | 0.033907000 | -1.141632000 | 0.723540000 |
| C | 0.593939000 | -2.499565000 | -0.830828000 |
| H | 1.657557000 | -2.578670000 | -0.605696000 |
| H | 0.477725000 | -2.572204000 | -1.916570000 |
| C | -1.398654000 | -0.954885000 | -0.835827000 |
| H | -1.419957000 | -0.856275000 | -1.925622000 |
| C | -2.197267000 | -2.185093000 | -0.470946000 |
| H | -3.276148000 | -2.068537000 | -0.471953000 |
| C | -1.659012000 | -3.355524000 | -0.148786000 |
| H | -2.309952000 | -4.179562000 | 0.123543000 |
| C | -0.180839000 | -3.618410000 | -0.145389000 |
| H | 0.021168000 | -4.572188000 | -0.637879000 |
| H | 0.165951000 | -3.732347000 | 0.887490000 |
| C | -2.008242000 | 0.304205000 | -0.256029000 |
| C | -2.233683000 | 1.421117000 | -1.055213000 |
| C | -2.330249000 | 0.374316000 | 1.098105000 |
| C | -2.766725000 | 2.586126000 | -0.515472000 |
| H | -1.992677000 | 1.378945000 | -2.111671000 |
| C | -2.862879000 | 1.534191000 | 1.640924000 |
| H | -2.157719000 | -0.488995000 | 1.731539000 |
| C | -3.082134000 | 2.645471000 | 0.834496000 |
| H | -2.938141000 | 3.444915000 | -1.152156000 |
| H | -3.107873000 | 1.572360000 | 2.694893000 |
| H | -3.498545000 | 3.550980000 | 1.256823000 |
| B | 1.934980000 | 0.097753000 | 1.591406000 |
| F | 2.185020000 | -1.261907000 | 1.600886000 |
| F | 2.984747000 | 0.797457000 | 2.149263000 |
| F | 0.743188000 | 0.409853000 | 2.212999000 |

**2′-BF$_3$-endo**
***E* =** -1037.089136
***H* =** -1037.069828
***G* =** -1037.138358
***N*$_{imag}$ =** 0
***SP* =** -1035.687834974835

| | | | |
|---|---|---|---|
| C | -3.832551000 | 1.020036000 | -0.763289000 |
| C | -3.782192000 | 2.387904000 | -0.638646000 |
| C | -2.653109000 | 2.956154000 | -0.067697000 |
| C | -1.626847000 | 2.132647000 | 0.351301000 |
| C | -1.720201000 | 0.753554000 | 0.204140000 |
| N | -2.829276000 | 0.223882000 | -0.352640000 |
| H | -2.574285000 | 4.028919000 | 0.049061000 |
| H | -4.681778000 | 0.516456000 | -1.197867000 |
| H | -4.612312000 | 2.987163000 | -0.981435000 |
| H | -0.736920000 | 2.551833000 | 0.798875000 |
| C | -0.589477000 | -0.127897000 | 0.666622000 |
| H | -0.800902000 | -1.160088000 | 0.403339000 |
| C | 0.732462000 | 0.252487000 | -0.011453000 |
| H | 1.067135000 | 1.233806000 | 0.338312000 |
| H | 0.605808000 | 0.313664000 | -1.094381000 |
| C | -0.432733000 | -0.065924000 | 2.191156000 |
| H | -0.428151000 | 0.973509000 | 2.536373000 |
| H | -1.294417000 | -0.541814000 | 2.665402000 |
| C | 0.831200000 | -0.748723000 | 2.630209000 |
| H | 0.916293000 | -0.986279000 | 3.685181000 |
| C | 1.816589000 | -1.081645000 | 1.804872000 |

| | | | |
|---|---|---|---|
| H | 2.689598000 | -1.596840000 | 2.193044000 |
| C | 1.811878000 | -0.783758000 | 0.326194000 |
| B | -3.007322000 | -1.382330000 | -0.549332000 |
| F | -2.913485000 | -1.964580000 | 0.700358000 |
| F | -2.004040000 | -1.810446000 | -1.398376000 |
| F | -4.251947000 | -1.593875000 | -1.104150000 |
| C | 3.162391000 | -0.316188000 | -0.172570000 |
| C | 3.926298000 | 0.584500000 | 0.567515000 |
| C | 3.645033000 | -0.744352000 | -1.406264000 |
| C | 5.145293000 | 1.043598000 | 0.087387000 |
| H | 3.562693000 | 0.926459000 | 1.530376000 |
| C | 4.864028000 | -0.286945000 | -1.891181000 |
| H | 3.060390000 | -1.445713000 | -1.991080000 |
| C | 5.618301000 | 0.608482000 | -1.144763000 |
| H | 5.728409000 | 1.739949000 | 0.676815000 |
| H | 5.225974000 | -0.633918000 | -2.850803000 |
| H | 6.570056000 | 0.963531000 | -1.518680000 |
| H | 1.571028000 | -1.709625000 | -0.209086000 |

**2′-BF$_3$-exo**
***E* =** -1037.080851
***H* =** -1037.062558
***G* =** -1037.129298
**$N_{imag}$ =** 0

| | | | |
|---|---|---|---|
| C | -3.755293000 | 1.371809000 | -0.370874000 |
| C | -3.478098000 | 2.658449000 | 0.023262000 |
| C | -2.217102000 | 2.934867000 | 0.532082000 |
| C | -1.292218000 | 1.914129000 | 0.618694000 |
| C | -1.615667000 | 0.626026000 | 0.206009000 |
| N | -2.849545000 | 0.380344000 | -0.279799000 |
| H | -1.959271000 | 3.934593000 | 0.855459000 |
| H | -4.715616000 | 1.091173000 | -0.774079000 |
| H | -4.236480000 | 3.421182000 | -0.070778000 |
| H | -0.301434000 | 2.101966000 | 1.010157000 |
| C | -0.604300000 | -0.482002000 | 0.353908000 |
| H | -0.955442000 | -1.357777000 | -0.178457000 |
| C | 0.779352000 | -0.097447000 | -0.231830000 |
| H | 0.758356000 | 0.897113000 | -0.679696000 |
| H | 1.018419000 | -0.794589000 | -1.036163000 |
| C | -0.521645000 | -0.882352000 | 1.850433000 |
| C | 1.930292000 | -0.157439000 | 0.800078000 |
| B | -3.298111000 | -1.102362000 | -0.784021000 |
| F | -3.152578000 | -1.967434000 | 0.283313000 |
| F | -2.480821000 | -1.445602000 | -1.844167000 |
| F | -4.616225000 | -1.018433000 | -1.180193000 |
| C | 3.257744000 | 0.050419000 | 0.114825000 |
| C | 3.903752000 | 1.281273000 | 0.180574000 |
| C | 3.844136000 | -0.972242000 | -0.629492000 |
| C | 5.110911000 | 1.488782000 | -0.475662000 |
| H | 3.457551000 | 2.084968000 | 0.755681000 |
| C | 5.050290000 | -0.770120000 | -1.286037000 |
| H | 3.351589000 | -1.936529000 | -0.696230000 |
| C | 5.688267000 | 0.462311000 | -1.210337000 |
| H | 5.600986000 | 2.451967000 | -0.409026000 |
| H | 5.494121000 | -1.575933000 | -1.857066000 |
| H | 6.630422000 | 0.619651000 | -1.719375000 |
| C | 1.830961000 | -1.442048000 | 1.581274000 |
| C | 0.652001000 | -1.787086000 | 2.085267000 |
| H | -0.404035000 | 0.025711000 | 2.453570000 |
| H | -1.460941000 | -1.345967000 | 2.147380000 |
| H | 0.522781000 | -2.693380000 | 2.664079000 |

| | | | |
|---|---|---|---|
| H | 2.718003000 | -2.047982000 | 1.726850000 |
| H | 1.784683000 | 0.672485000 | 1.502753000 |

**4-BF$_3$**
***E* =** -1037.082818
***H* =** -1037.063570
***G* =** -1037.131299
**$N_{imag}$ =** 0

| | | | |
|---|---|---|---|
| C | -1.779565000 | -0.025536000 | -1.217047000 |
| N | -2.301159000 | -0.653512000 | -0.154615000 |
| C | -1.515204000 | -1.402181000 | 0.627150000 |
| C | -0.168712000 | -1.542729000 | 0.370371000 |
| C | 0.402701000 | -0.885229000 | -0.717362000 |
| C | -0.441986000 | -0.120966000 | -1.521619000 |
| H | -1.999116000 | -1.882295000 | 1.464870000 |
| H | 0.430726000 | -2.151035000 | 1.033060000 |
| C | 1.883850000 | -0.916116000 | -0.982673000 |
| H | 2.039020000 | -0.478762000 | -1.969765000 |
| C | 2.469661000 | -2.347631000 | -0.958146000 |
| H | 2.697674000 | -2.658383000 | -1.977546000 |
| H | 1.721052000 | -3.045665000 | -0.582532000 |
| C | 2.629340000 | 0.043032000 | 0.025987000 |
| C | 2.918483000 | -0.665452000 | 1.320609000 |
| H | 2.715816000 | -0.171403000 | 2.263024000 |
| C | 3.471918000 | -1.872356000 | 1.268168000 |
| H | 3.731324000 | -2.414537000 | 2.169532000 |
| C | 3.722685000 | -2.485023000 | -0.077724000 |
| H | 3.999810000 | -3.534825000 | 0.005156000 |
| H | 4.560966000 | -1.972314000 | -0.562695000 |
| H | 3.591638000 | 0.235711000 | -0.461563000 |
| C | 1.916150000 | 1.367954000 | 0.154089000 |
| C | 2.137128000 | 2.361831000 | -0.796982000 |
| C | 0.974391000 | 1.603816000 | 1.154213000 |
| C | 1.434051000 | 3.558585000 | -0.756369000 |
| H | 2.868316000 | 2.193743000 | -1.580422000 |
| C | 0.270031000 | 2.799740000 | 1.200094000 |
| H | 0.773621000 | 0.842575000 | 1.898883000 |
| C | 0.495438000 | 3.780652000 | 0.243281000 |
| H | 1.620432000 | 4.318593000 | -1.504544000 |
| H | -0.459965000 | 2.962503000 | 1.982917000 |
| H | -0.054664000 | 4.712070000 | 0.277602000 |
| H | -0.054965000 | 0.412014000 | -2.379441000 |
| H | -2.468923000 | 0.559269000 | -1.810760000 |
| B | -3.870263000 | -0.451109000 | 0.152572000 |
| F | -4.553249000 | -0.796644000 | -0.998113000 |
| F | -4.193565000 | -1.268428000 | 1.214560000 |
| F | -4.050464000 | 0.886018000 | 0.454378000 |

**4-H**
***E* =** -712.866093
***H* =** -712.850836
***G* =** -712.908342
**$N_{imag}$ =** 0

| | | | |
|---|---|---|---|
| C | 1.418182000 | 2.566134000 | -0.851615000 |
| N | 1.107918000 | 3.188744000 | 0.293548000 |
| C | 0.049336000 | 2.843348000 | 1.040171000 |
| C | -0.761811000 | 1.813503000 | 0.634374000 |
| C | -0.475356000 | 1.123090000 | -0.546080000 |
| C | 0.636011000 | 1.525909000 | -1.288206000 |
| H | -0.109805000 | 3.408304000 | 1.946150000 |
| H | -1.607541000 | 1.542182000 | 1.249928000 |

| | | | |
|---|---|---|---|
| C | -1.279790000 | -0.069796000 | -0.979977000 |
| H | -0.965253000 | -0.313202000 | -1.995068000 |
| C | -2.803020000 | 0.196627000 | -0.965062000 |
| H | -3.153478000 | 0.320317000 | -1.989311000 |
| H | -3.010711000 | 1.136146000 | -0.452013000 |
| C | -0.887081000 | -1.324292000 | -0.102544000 |
| C | -1.693498000 | -1.364140000 | 1.166159000 |
| H | -1.205283000 | -1.575826000 | 2.109344000 |
| C | -3.009140000 | -1.195476000 | 1.081402000 |
| H | -3.642624000 | -1.249481000 | 1.958490000 |
| C | -3.607645000 | -0.908465000 | -0.263359000 |
| H | -4.650727000 | -0.607223000 | -0.182129000 |
| H | -3.584920000 | -1.815573000 | -0.877485000 |
| H | -1.205511000 | -2.173646000 | -0.717107000 |
| C | 0.610435000 | -1.428316000 | 0.064728000 |
| C | 1.374337000 | -1.975468000 | -0.964214000 |
| C | 1.268429000 | -0.920102000 | 1.182722000 |
| C | 2.759676000 | -2.011171000 | -0.884101000 |
| H | 0.875848000 | -2.377180000 | -1.839853000 |
| C | 2.654659000 | -0.956540000 | 1.268548000 |
| H | 0.700765000 | -0.480495000 | 1.994345000 |
| C | 3.405153000 | -1.499236000 | 0.234423000 |
| H | 3.334543000 | -2.442982000 | -1.693484000 |
| H | 3.147582000 | -0.557269000 | 2.145845000 |
| H | 4.485027000 | -1.527080000 | 0.301203000 |
| H | 0.899618000 | 1.019208000 | -2.205704000 |
| H | 2.289573000 | 2.924359000 | -1.378422000 |
| H | 1.694206000 | 3.954580000 | 0.606226000 |

**4-COMe**
***E* =** -865.466465
***H* =** -865.447820
***G* =** -865.513304
**$N_{imag}$ =** 0

| | | | |
|---|---|---|---|
| C | 2.048591000 | -0.042182000 | 1.152667000 |
| N | 2.527175000 | -0.765646000 | 0.119333000 |
| C | 1.704628000 | -1.543843000 | -0.616141000 |
| C | 0.368412000 | -1.613673000 | -0.330352000 |
| C | -0.164468000 | -0.868395000 | 0.726380000 |
| C | 0.716681000 | -0.079971000 | 1.469049000 |
| H | 2.173999000 | -2.085112000 | -1.423129000 |
| H | -0.261212000 | -2.239847000 | -0.945665000 |
| C | -1.635077000 | -0.845478000 | 1.018548000 |
| H | -1.761558000 | -0.353449000 | 1.983092000 |
| C | -2.259628000 | -2.260544000 | 1.071154000 |
| H | -2.517992000 | -2.495591000 | 2.103061000 |
| H | -1.523072000 | -3.002911000 | 0.762272000 |
| C | -2.363392000 | 0.083432000 | -0.033196000 |
| C | -2.652371000 | -0.675838000 | -1.297777000 |
| H | -2.436279000 | -0.226331000 | -2.259064000 |
| C | -3.222107000 | -1.872227000 | -1.195807000 |
| H | -3.482080000 | -2.449981000 | -2.074474000 |
| C | -3.498295000 | -2.418578000 | 0.173295000 |
| H | -3.790768000 | -3.466595000 | 0.135764000 |
| H | -4.335288000 | -1.871036000 | 0.620691000 |
| H | -3.324166000 | 0.300785000 | 0.446499000 |
| C | -1.635626000 | 1.396302000 | -0.200925000 |
| C | -1.839783000 | 2.410210000 | 0.733101000 |
| C | -0.704010000 | 1.605261000 | -1.216098000 |
| C | -1.130540000 | 3.601090000 | 0.661248000 |
| H | -2.564761000 | 2.262808000 | 1.526201000 |

| | | | |
|---|---|---|---|
| C | 0.006256000 | 2.796836000 | -1.293140000 |
| H | -0.518579000 | 0.831923000 | -1.952434000 |
| C | -0.202530000 | 3.797599000 | -0.353656000 |
| H | -1.305014000 | 4.376946000 | 1.395695000 |
| H | 0.725764000 | 2.941418000 | -2.088982000 |
| H | 0.350917000 | 4.725707000 | -0.413848000 |
| H | 0.362383000 | 0.517853000 | 2.296967000 |
| H | 2.751374000 | 0.558324000 | 1.706824000 |
| C | 3.970783000 | -0.742312000 | -0.257305000 |
| O | 4.307591000 | -1.452999000 | -1.144310000 |
| C | 4.844322000 | 0.180709000 | 0.519391000 |
| H | 4.868664000 | -0.108711000 | 1.570433000 |
| H | 5.842750000 | 0.110572000 | 0.100960000 |
| H | 4.482900000 | 1.206366000 | 0.445665000 |
